# The combination of the tubulin binding small molecule PTC596 and proteasome inhibitors suppresses the growth of myeloma cells

**DOI:** 10.1038/s41598-021-81577-x

**Published:** 2021-01-22

**Authors:** Yurie Nagai, Naoya Mimura, Ola Rizq, Yusuke Isshiki, Motohiko Oshima, Mohamed Rizk, Atsunori Saraya, Shuhei Koide, Yaeko Nakajima-Takagi, Makiko Miyota, Tetsuhiro Chiba, Nagisa Oshima-Hasegawa, Tomoya Muto, Shokichi Tsukamoto, Shio Mitsukawa, Yusuke Takeda, Chikako Ohwada, Masahiro Takeuchi, Tohru Iseki, Chiaki Nakaseko, William Lennox, Josephine Sheedy, Marla Weetall, Koutaro Yokote, Atsushi Iwama, Emiko Sakaida

**Affiliations:** 1 Department of Hematology, Chiba University Hospital, Chiba, Japan; 2 Department of Endocrinology, Hematology, and Gerontology, Chiba University Graduate School of Medicine, Chiba, Japan; 3 Department of Transfusion Medicine and Cell Therapy, Chiba University Hospital, 1-8-1 Inohana, Chuo-ku, Chiba, 260-8677 Japan; 4 Division of Stem Cell and Molecular Medicine, Center for Stem Cell Biology and Regenerative Medicine, The Institute of Medical Science, The University of Tokyo, 4-6-1 Shirokanedai, Minato-ku, Tokyo, 108-8639 Japan; 5 Department of Cellular and Molecular Medicine, Chiba University Graduate School of Medicine, Chiba, Japan; 6 Department of Gastroenterology, Graduate School of Medicine, Chiba University, Chiba, Japan; 7 Department of Hematology, International University of Health and Welfare School of Medicine, Narita, Japan; 8 PTC Therapeutics Inc., South Plainfield, NJ USA

**Keywords:** Myeloma, Targeted therapies

## Abstract

The novel small molecule PTC596 inhibits microtubule polymerization and its clinical development has been initiated for some solid cancers. We herein investigated the preclinical efficacy of PTC596 alone and in combination with proteasome inhibitors in the treatment of multiple myeloma (MM). PTC596 inhibited the proliferation of MM cell lines as well as primary MM samples in vitro, and this was confirmed with MM cell lines in vivo. PTC596 synergized with bortezomib or carfilzomib to inhibit the growth of MM cells in vitro. The combination treatment of PTC596 with bortezomib exerted synergistic effects in a xenograft model of human MM cell lines in immunodeficient mice and exhibited acceptable tolerability. Mechanistically, treatment with PTC596 induced cell cycle arrest at G2/M phase followed by apoptotic cell death, associated with the inhibition of microtubule polymerization. RNA sequence analysis also revealed that PTC596 and the combination with bortezomib affected the cell cycle and apoptosis in MM cells. Importantly, endoplasmic reticulum stress induced by bortezomib was enhanced by PTC596, providing an underlying mechanism of action of the combination therapy. Our results indicate that PTC596 alone and in combination with proteasome inhibition are potential novel therapeutic options to improve outcomes in patients with MM.

## Introduction

Over the past decade, the prognosis of multiple myeloma (MM) has improved dramatically due to the introduction of proteasome inhibitors and immunomodulatory drugs (IMiDs). Recently, monoclonal antibodies and new drugs targeting epigenetic regulation have also been added to the therapeutic options to improve patient outcome. However, MM remains an incurable disease associated with complex heterogeneity; therefore, innovative therapeutic strategies of multiple drug combination are needed to conquer it^1^.

Proteasome inhibitors have dramatically changed the treatment strategies for multiple myeloma. Bortezomib, the first approved proteasome inhibitor, is widely used in combination therapies because of its effectiveness, tolerability, and combinability^2^. Bortezomib has various modes of action including induction of endoplasmic reticulum (ER) stress, upregulation of pro-apoptotic proteins, suppression of anti-apoptotic proteins, inhibition of NF-κB and its downstream anti-apoptotic genes, and dysregulation of the DNA repair pathway^3^; and therefore, it can be currently combined with a variety of drugs such as alkylating reagents, IMiDs^4^, monoclonal anti-CD38 antibodies^5^, and a histone deacetylase inhibitor^6^. Moreover, next-generation proteasome inhibitors carfilzomib and ixazomib are also available in the clinical practice. To increase therapeutic options for patients, clinical trials of the novel combination of bortezomib with a BCL-2 inhibitor, an XPO1 inhibitor, or a monoclonal anti-BCMA antibody are ongoing in MM (NCT02755597, NCT03110562, NCT04091126). In the preclinical stage, we have shown proteasome inhibitors-containing combination strategies using an IRE1α endoribonuclease domain inhibitor, a selective Akt inhibitor, and an EZH2/EZH1 dual inhibitor in the treatment of MM^7–9^.

PTC596 was originally developed to target cancer stem cells with degradation of B-cell specific Moloney murine leukemia virus integration site 1 (BMI1), which is a component of PRC1 that maintains transcriptional repression of target genes via ubiquityl histone H2A (uH2A)^10^. Recently, PTC596 has been recognized as a direct microtubule polymerization inhibitor in a preclinical study of pancreatic ductal adenocarcinoma^11^. PTC596 induces cytotoxicity with EC_50_ values of 30–200 nM in various tumor cell lines^12^ and has preclinical effects on hematological malignancies such as acute myeloid leukemia and mantle cell leukemia^13,14^. Clinical trials of PTC596 are ongoing for glioma, leiomyosarcoma, and ovarian cancer. (NCT03605550, NCT03761095, NCT03206645). More recently, PTC596 has shown activity killing MM cells, alone and in combination with BH3 mimetics or epigenetic modulators^15^. In the current study, we focused on the preclinical activities and modes of action of PTC596 alone as well as in combination with proteasome inhibitors in MM.

## Results

### PTC596 induces cytotoxicity in MM cells

**Figure 1:**
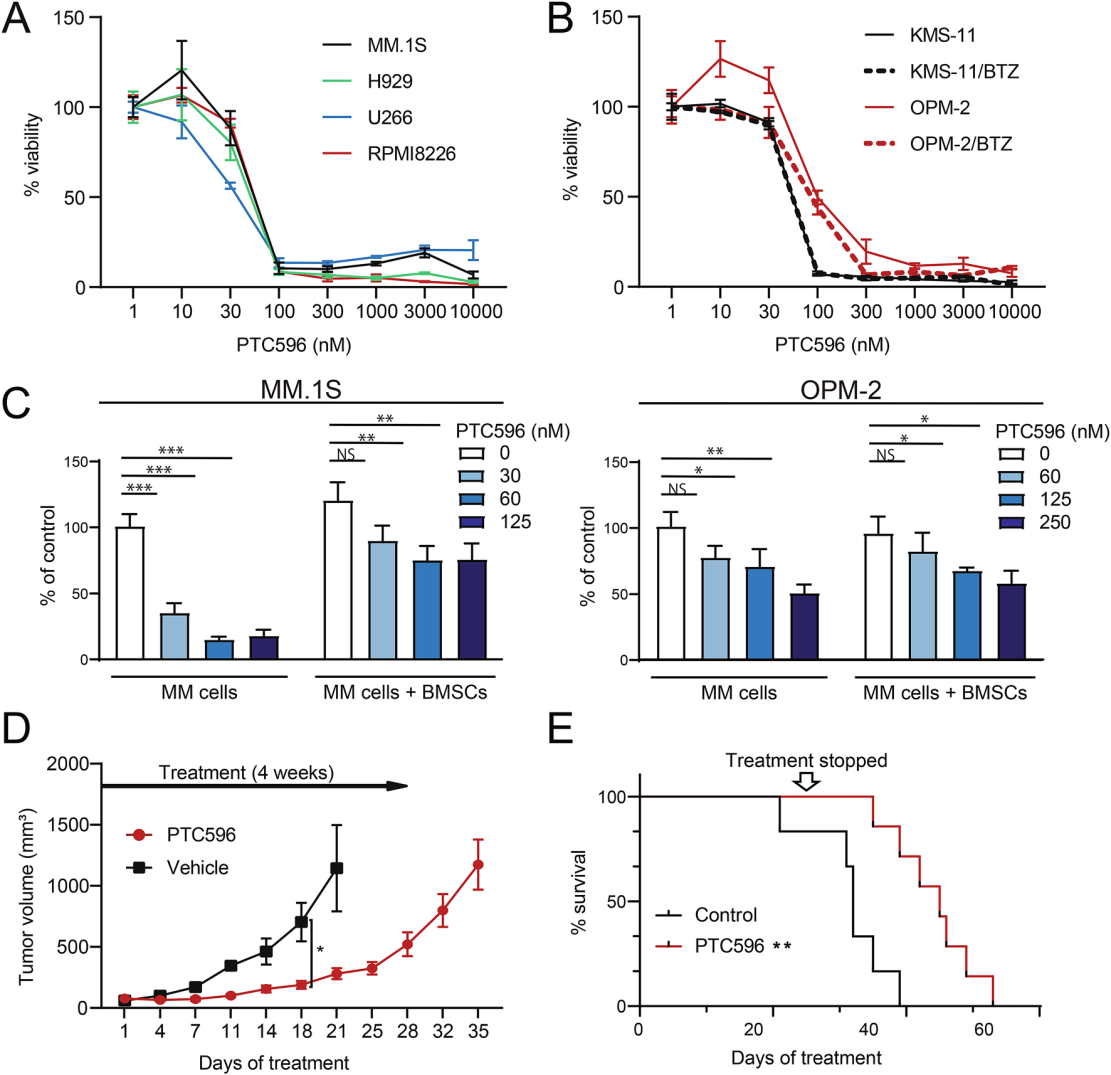
PTC596 inhibits the growth of MM cells both in vitro and in vivo. (**A**, **B**) MTS assays of (**A**) MM.1S, H929, RPMI8226, U266, and (**B**) KMS-11, KMS-11/BTZ, OPM-2, OPM-2/BTZ treated with the indicated doses of PTC596 for 72 h. The y-axis presents percent viability relative to the untreated control. Data are shown as means ± SD of triplicate or quadruplicate samples. (**C**) Cell proliferation assays evaluated by BrdU incorporation of MM.1S and OPM2 cells co-cultured with or without BMSCs isolated from patients with MM upon treatment with the indicated doses of PTC596 for 48 h. BrdU was added to the culture 2 h before the analysis. Y-axis is presented as proliferation rate relative to an untreated control. Data are shown as mean ± SD of triplicate samples. *P < 0.05; **P < 0.01; ***P < 0.001; ns, not significant by a one-way ANOVA. (**D**, **E**) In vivo analysis of the cytotoxicity of PTC596 using a murine xenograft model of human myeloma MM.1S cells. NOG mice were injected subcutaneously on the right side of the back with 4 × 10^6^ MM.1S cells. After the tumor grew to a measurable size, treatment was initiated. Mice were treated with oral PTC596 (12.5 mg/kg) (n = 7) or control vehicle (n = 6) twice a week for 4 weeks. (**D**) Tumor volumes were monitored twice a week. Data represent mean ± SEM. *P < 0.05 using Student’s *t*-test. (**E**) Kaplan–Meier survival of mice. The mice were sacrificed when the tumors reached 2000 cm^3^ or an ulcer occurred. Survival was evaluated from the first day of treatment to death. The statistical significance of differences between PTC596-treated and vehicle-treated groups was determined using a log-rank test. **P < 0.01.

We first examined the cytotoxic effect of PTC596 alone on MM cells. PTC596 induced significant cytotoxicity in several MM cell lines, including bortezomib-resistant lines such as KMS-11/BTZ, and OMP-2/BTZ in MTS assays (Fig. 1A,B). PTC596 exhibited cytotoxicity against MM cells irrespective of *TP53* status (*TP53* wild-type: MM.1S, H929; *TP53* mutation: RPMI8226, U266, OPM-2, OPM-2/BTZ; *TP53* deletion: KMS-11, KMS-11/BTZ according to the IARC TP53 database^16^). The concentrations of PTC596 required to inhibit cell viability by 50% (cytotoxic concentration; CC_50_) were quite low against all cell lines tested, ranging from 25 to 100 nM (Supplementary Table S1). We also evaluated the efficacy of PTC596 in MM cell lines co-cultured with bone marrow stromal cells (BMSCs) from patients with MM by BrdU proliferation assays. As reported^17^, MM cells grew better when co-cultured with BMSCs than without BMSCs. PTC596 suppressed the proliferation of MM cells even in the presence of BMSCs (Fig. 1C).

In order to determine the efficacy of PTC596 in MM cells in vivo, we next generated subcutaneous xenograft models of MM.1S cells in NOG immunodeficient mice. PTC596 (12.5 mg/kg) or control vehicle was administered orally twice a week for 4 weeks. PTC596 significantly inhibited the growth of tumors implanted in mice (p = 0.022 on day 18, p = 0.058 on day 21; Fig. 1D) and prolonged the survival of mice as compared with the control (p = 0.0021; Fig. 1E). PTC596 treatment was well tolerated with slight weight loss and mild diarrhea which immediately recovered after the end of treatment.

### PTC596 inhibits microtubule polymerization and triggers cell cycle arrest in MM cells

**Figure 2:**
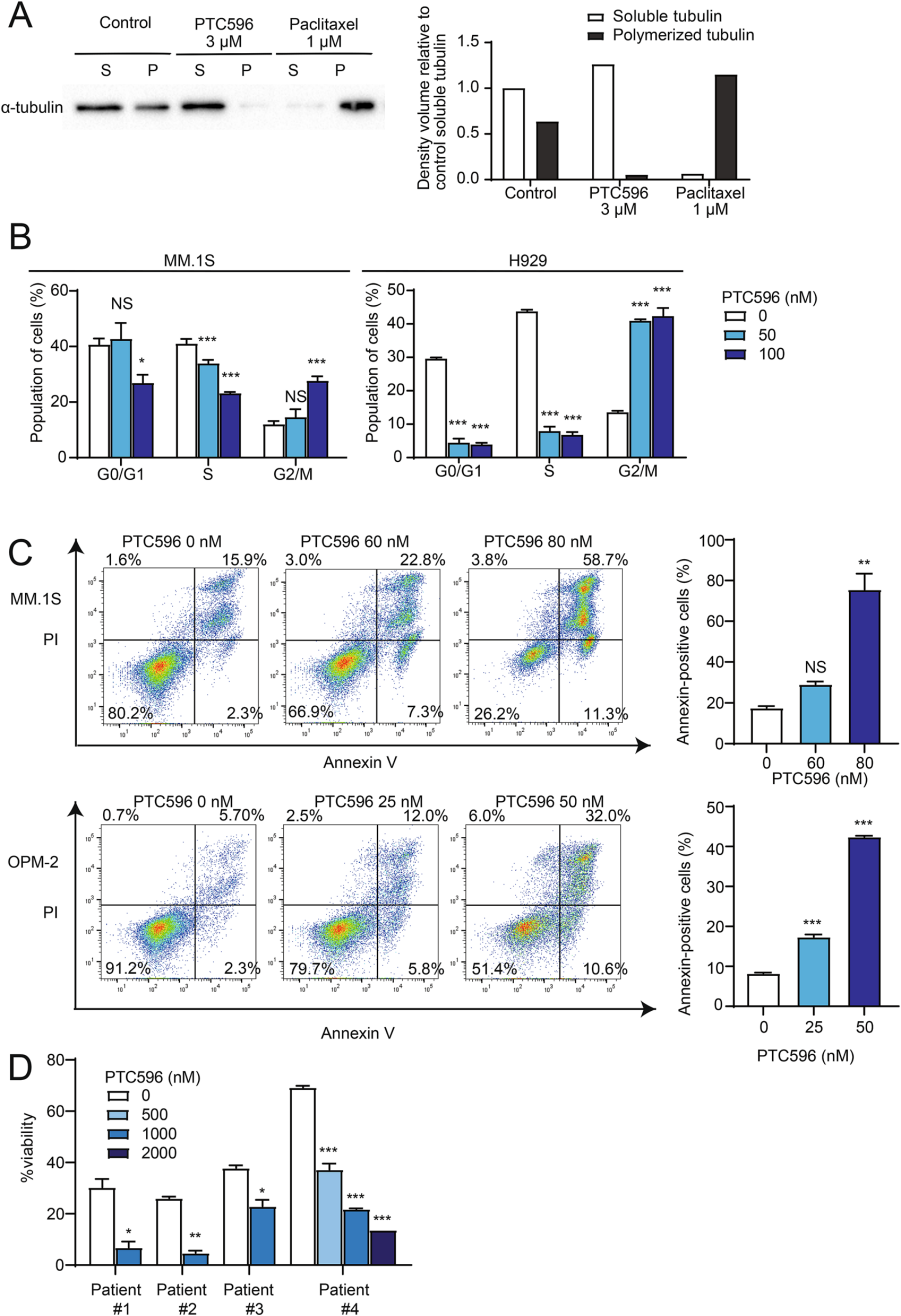
PTC596 induces cell cycle arrest and apoptosis associated with the inhibition of microtubule polymerization. (**A**) Western blotting analysis of soluble and polymerized microtubules in MM cells detected using an anti-α-tubulin antibody. MM.1S were incubated in the presence or absence of PTC596 or paclitaxel for 4 h. After treatment, cell lysates were fractionated by centrifugation to separate free tubulin (soluble tubulin) from microtubules (polymerized tubulin). The graphs show the density volumes of α-tubulin normalized to that of the soluble fraction in the control. (**B**) Cell cycle analysis of MM.1S and H929 treated with the indicated doses of PTC596 for 24 h, exposed to BrdU for 2 h, followed by flow cytometric analyses. Data represent mean ± SD of triplicate experiments. *P < 0.05; ***P < 0.001; ns, not significant using one-way ANOVA. (**C**) Annexin V staining of MM.1S and OPM-2 treated with the indicated doses of PTC596 for 48 h. Apoptotic cells were detected as Annexin V-positive cells by flow cytometry. The representative flow cytometric profiles are shown in the left panels, and the results of duplicate experiments are shown in the right graphs. Data represent mean ± SD. **P < 0.01; ***P < 0.001; ns, not significant using one-way ANOVA. (**D**) The cytotoxicity of the indicated doses of PTC596 against primary MM cells analyzed by Annexin V staining using flow cytometry. Primary MM cells were treated for 12–24 h. Data represent mean ± SD of duplicate experiments. *P < 0.05; **P < 0.01; ***P < 0.001 using Student’s *t*-test or one-way ANOVA.

PTC596 has recently been demonstrated to directly inhibit microtubule polymerization in pancreatic ductal adenocarcinoma^11^. Therefore, we investigated the effects of PTC596 on the levels of soluble (unpolymerized) versus polymerized tubulin content in MM cells using protein lysates from vehicle- or PTC596-treated MM.1S cells with tubulin preservation buffer. Visualization of tubulin fractions by western blotting demonstrated that PTC596 treatment led to a near-complete loss of polymerized microtubules. In contrast, polymerized microtubules were increased in cells treated with paclitaxel, which stabilizes the microtubule polymer and protects it from disassembly^18^ (Fig. 2A). These results supported the inhibitory function of PTC596 against microtubule polymerization.

Because microtubules play a critical role in the separation of chromosomes during mitosis, inhibition of microtubule polymerization results in mitotic arrest. Overnight treatment with PTC596 significantly increased MM cells in G2/M phase, suggesting that PTC596 induced G2/M cell cycle arrest (Fig. 2B). Annexin V staining also demonstrated that PTC596 treatment for 2 days induced massive apoptosis in MM cell lines in a dose-dependent manner (Fig. 2C). These results suggested that the cytotoxicity of PTC596 in MM cells is provoked by mitotic arrest and subsequent apoptosis.

Next, we evaluated the efficacy of PTC596 in primary MM cells derived from patients. We purified CD138^+^ cells from the bone marrow (BM) of patients with MM and cultured them in the presence of PTC596 for 12–24 h. Of note, PTC596 treatment significantly reduced the proportion of Annexin V-negative viable MM cells in culture (Fig. 2D).

### PTC596 in combination with proteasome inhibitors exerts augmented cytotoxicity against MM cells

**Figure 3:**
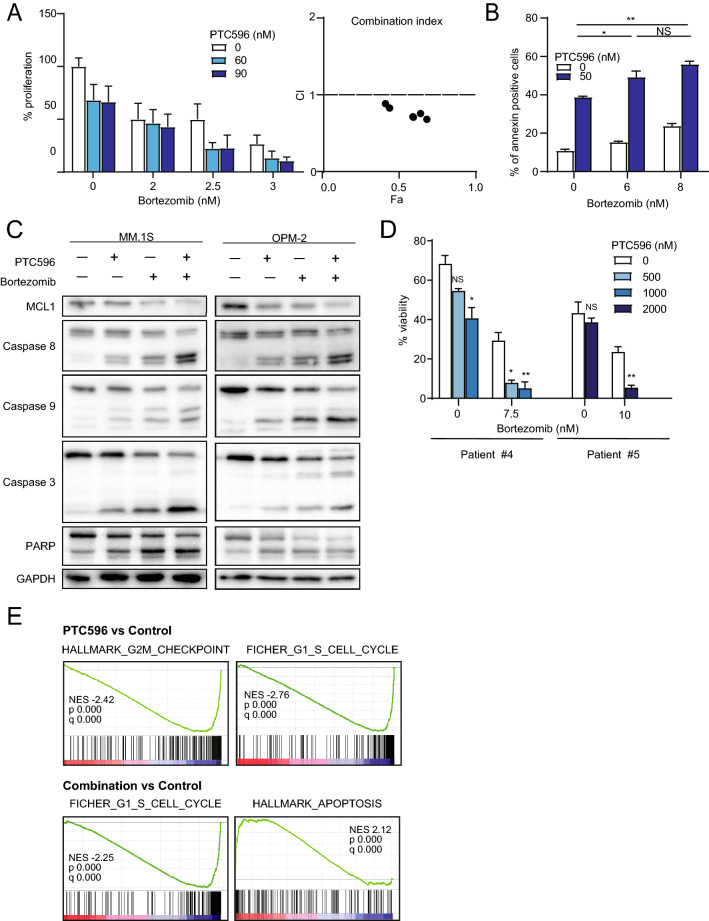
PTC596 enhances bortezomib-induced apoptosis. (**A**) BrdU proliferation assay of MM.1S co-cultured with BMSCs derived from patients with MM upon treatment with the indicated doses of PTC596 and bortezomib for 48 h. Results of triplicate experiments and combination index values are shown in the left and right graphs, respectively. (**B**) Annexin V staining of OPM-2 treated with the indicated doses of PTC596 for 48 h and bortezomib for 24 h. Apoptotic cells were detected as Annexin V-positive cells by flow cytometry, and the results of duplicate experiments are shown. Data represent mean ± SD. *P < 0.05; **P < 0.01; ns, not significant using one-way ANOVA. (**C**) Western blotting analysis of the indicated proteins in MM.1S and OPM-2. MM.1S and OPM-2 were treated with PTC596 (100 nM) and/or bortezomib (2 nM for MM.1S and 5 nM for OPM-2) for 48 h. GAPDH served as a loading control. (**D**) The cytotoxicity of the combination treatment of PTC596 and bortezomib against primary MM cells. Viable cells were defined as those negative for Annexin V and PI by flow cytometric analyses. Primary MM cells were treated with the indicated doses of PTC596 and/or bortezomib for 12 or 24 h. Data represent mean ± SD. *P < 0.05; **P < 0.01; n.s., not significant using Student’s *t*-test or one-way ANOVA. (**E**) Gene set enrichment analysis plots showing the enrichment of cell cycle or apoptosis gene sets in MM.1S treated with PTC596 alone (left panels) and the combination (right panels) compared with non-treated control cells. MM.1S cells were treated with PTC596 (100 nM) alone or in combination with bortezomib (2 nM) for 24 h. Total RNA was extracted and RNA-seq was performed. Normalized enrichment scores (NES), nominal p values (NOM), and false discovery rates (FDR) are indicated.

To enhance the therapeutic benefits of PTC596 in MM cells, we investigated the possible synergism between PTC596 and the proteasome inhibitor bortezomib, which is a first-line therapeutic agent in the treatment of MM. We treated MM.1S cells with increasing concentrations of PTC596 (0 to 90 nM) and bortezomib (0 to 3 nM) as a single agent or in combination. After 2 days of culture, cells were analyzed using MTS assays. PTC596 and bortezomib did not show a synergistic cytotoxic effect on MM cells in suspension culture, showing a high combination index (CI), which defines synergism when the calculated number is less than 1 (Supplementary Fig. S1A, Supplementary Table S2B)^19^. In contrast, PTC596 and bortezomib exerted synergistic or additive cytotoxic effects with a CI less than or around 1 when MM cells were co-cultured with BMSCs derived from patients with MM, which mimics the BM microenvironment (Fig. 3A, Supplementary Fig. S2A, Supplementary Table S2A,C). PTC596 and carfilzomib, another proteasome inhibiter, also showed synergistic or additive cytotoxic effects on MM cells when those were co-cultured with BMSCs (Supplementary Fig. S3A, Supplementary Table S2D). We next performed Annexin V staining to evaluate apoptosis in the combination treatment. Apoptosis induced by PTC596 (50 nM) was significantly augmented by the combination with bortezomib (Fig. 3B). Enhanced apoptosis in the combination was confirmed by western blotting; the combination with PTC596 and bortezomib enhanced cleavage of caspases, which was accompanied by a reduction in MCL1 protein levels compared with each single treatment (Fig. 3C). Notably, PTC596 in combination with bortezomib also induced significant cytotoxicity in primary CD138^+^ cells from the BM of patients with MM compared with bortezomib alone, as evidenced by reductions in Annexin V-negative viable cells (Fig. 3D).

To understand the effects of PTC596 on global transcriptional profiles in MM cells, we performed RNA-seq of MM.1S cells treated with PTC596 alone or in combination with bortezomib. Several gene sets related to cell cycle were negatively enriched in MM cells treated with PTC596 alone as well as PTC596 and bortezomib. In addition, apoptosis-related gene sets were positively enriched in MM cells treated with PTC596 and bortezomib (Fig. 3E and Supplementary Table S3–S6).

### PTC596 and bortezomib demonstrate synergistic effects in a xenograft model of human myeloma cells

**Figure 4:**
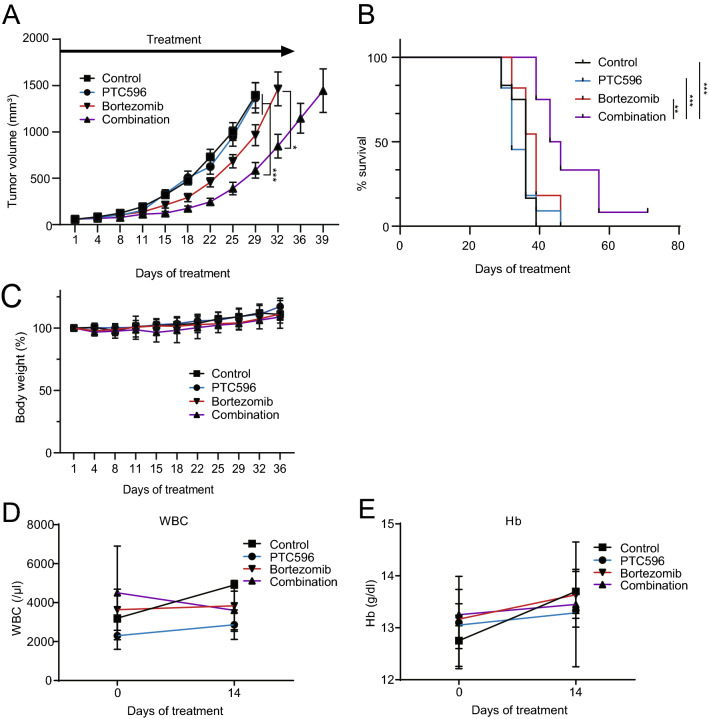
PTC596 and bortezomib exert synergistic anti-MM activity in a xenograft MM model. NOG mice were injected subcutaneously on the right side of the back with 4 × 10^6^ MM.1S cells. After the tumor grew to a measurable size, treatment was initiated. Mice were treated for 5 weeks with oral PTC596 (6.25 mg/kg) twice a week, subcutaneous bortezomib (0.5 mg/kg) in the left side of the back twice a week, or the combination. Control mice received orally administered vehicle and subcutaneous saline for 5 weeks. (**A**) Tumor volume was monitored twice a week in PTC596-treated group (n = 11), bortezomib-treated group (n = 11), combination group (n = 12), and vehicle-treated group (n = 12). Data represent mean ± SEM. *P < 0.05; ***P < 0.001 using one-way ANOVA and Student’s *t*-test. (**B**) Kaplan–Meier survival of mice. The mice were sacrificed when the tumors reached 2,000 cm^3^ or an ulcer occurred. Survival was evaluated from the first day of treatment to death. Statistical significance was determined using the log-rank test. **P < 0.01; ***P < 0.001. (**C**) Body weight of the mice. Data represent mean ± SD. (**D**) White blood cell counts and (**E**) Hemoglobin (Hb) of the mice examined on the indicated day of treatment. Data represent mean ± SD.

In order to evaluate the efficacy of the combination of PTC596 with bortezomib in vivo, we used subcutaneous xenograft models of MM.1S cells, as shown in Fig. 1D and E. PTC596 (6.25 mg/kg) was administered orally to mice in combination with subcutaneous injections of bortezomib (0.5 mg/kg) twice a week for 5 weeks. The combination therapy of PTC596 and bortezomib significantly reduced tumor growth compared with the control (day 29, p < 0.001), PTC596 alone (day 29, p < 0.001), and bortezomib alone (day 32, p = 0.010) (Fig. 4A). Survival of host mice treated with the combination was significantly prolonged compared with the control (p < 0.001), PTC596 alone (p < 0.001), and bortezomib alone (p = 0.007) (Fig. 4B). Of note, 1 out of 12 mice treated with combination therapy achieved a complete response with no residual tumor. Mice which received the combination therapy did not show significant body weight loss except one that needed a washout period of the drugs until recovery (Fig. 4C). Hematological toxicity was examined in another cohort, in which the mice were treated with or without oral PTC596 in combination with or without subcutaneous bortezomib for 2 weeks. White blood cell counts and hemoglobin levels in the peripheral blood of mice did not change significantly during treatment with PTC596 or even the combination (Fig. 4D, E). These results demonstrated that PTC596 in combination with bortezomib is effective and tolerable in vivo, holding promise of clinical applications.

### PTC596 functions independently of BMI1 in MM cells

**Figure 5:**
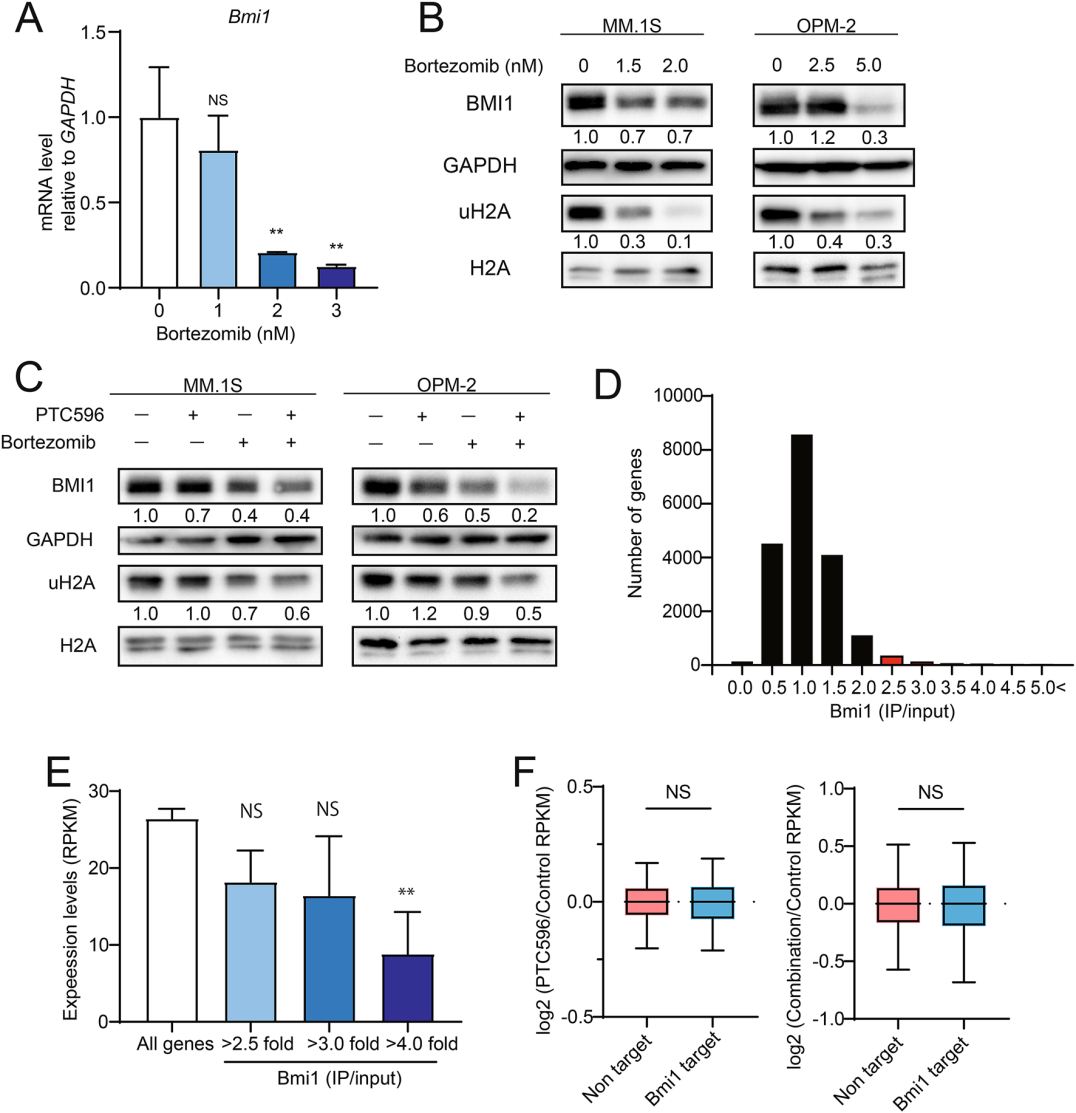
PTC596 does not directly target BMI1. (**A**) Quantitative RT-PCR analysis of *BMI1* in MM.1S treated with the indicated dose of bortezomib for 24 h. *GAPDH* was used to normalize the amount of input RNA. Data are shown as the mean ± SD (n = 3). **P < 0.01; n.s., not significant using one-way ANOVA. (**B**) Western blotting analysis of BMI1 and uH2A in MM.1S and OPM-2 upon treatment with the indicated doses of bortezomib for 48 h. GAPDH and H2A served as loading controls. (**C**) Western blotting analysis of BMI1 and uH2A in MM.1S and OPM-2 upon treatment with PTC596 (100 nM) and bortezomib (2 nM for MM.1S and 5 nM for OPM-2) alone or in combination for 48 h. GAPDH and H2A served as loading controls. (**D**) Bmi1 targets defined by ChIP-seq data of Bmi1. Graph showing the number of genes in each range of fold enrichment values (IP/input) of Bmi1. Genes with 2.5-fold enrichment of Bmi1 signals over the input signals in the promoter regions (transcription start sites ± 2.0 kb) were defined as direct targets of BMI1 (red bars). (**E**) Expression levels of all RefSeq genes and BMI1 target genes detected by RNA-seq (RPKM). Bmi1 target genes with fold enrichment greater than 2.5, 3.0, and 4.0 are depicted separately. **P < 0.01; n.s., not significant using Welch’s *t*-test. (**F**) Box-and-whisker plots showing the expression changes of 351 BMI1 target genes in MM.1S cells treated with PTC596 alone (left panel) and PTC596 in combination with bortezomib (right panel) compared with control cells. Boxes represent 25 to 75 percentile ranges. Vertical lines represent 10 to 90 percentile ranges. Horizontal bars represent median. *ns* not significant using Student’s *t*-test.

PTC596 was initially identified as an agent decreasing the levels of BMI1 protein^12,13^, which plays an oncogenic role in the maintenance of proliferative capacity of cells through repression of the *INK4a*/*ARF* pathway^20,21^. BMI1 becomes hyperphosphorylated and dissociates from chromatin during mitosis^22^, suggesting that PTC596 induces reductions in BMI1 protein levels as an indirect consequence of induction of mitotic arrest. The functional role of BMI1 in the activity of PTC596 has been tested in *Kras*/*p53* mutant pancreatic tumors, in which deletion of *Bmi1* did not affect the ability of PTC596 to inhibit cell proliferation^11^. Of interest, bortezomib was reported to repress the transcription of *BMI1* in the side population of mantle cell lymphoma cells^23^ and reduce the levels of mono-ubiquitination of histone H2A at Lysine 119 (uH2A)^24^. However, its impact on BMI1 in MM cells has not yet been elucidated. We examined *BMI1* mRNA levels by qPCR and the protein levels of BMI1 and uH2A by western blotting after bortezomib treatment in MM cells (Fig. 5A,B). Bortezomib significantly repressed the expression of *BMI1* and reduced the protein levels of BMI1 and uH2A. The combination treatment of PTC596 with bortezomib had additive effects on the levels of BMI1 and uH2A (Fig. 5C).

In order to evaluate the functional role of BMI1 in the activity of PTC596 alone and in combination with bortezomib, we determined BMI1 target genes in MM cells using MM.1S cells overexpressing mouse Bmi1 (mBmi1) by ChIP-seq as reported previously^25^. We defined 3517 genes with enrichment of Bmi1 signals over the input signals by 2.5-fold from the promoter regions (transcription start sites ± 2.0 kb) as direct targets of BMI1 (Fig. 5D). We then checked the correlation between expression levels and fold enrichment values (IP/input) of Bmi1 (Fig. 5E). The expression levels of these genes were inversely correlated with the fold enrichment values of Bmi1 (Fig. 5E). We then checked the expression levels of BMI1 targets after PTC596 treatment alone or in combination with bortezomib. Unexpectedly, PTC596 alone or in combination with bortezomib did not significantly de-repress the expression of BMI1 target genes in spite of significant reductions in BMI1 protein levels (Fig. 5F). These results supported the notion that PTC596 functions independently of BMI1.

### PTC596 enhanced endoplasmic reticulum stress induced by bortezomib

**Figure 6:**
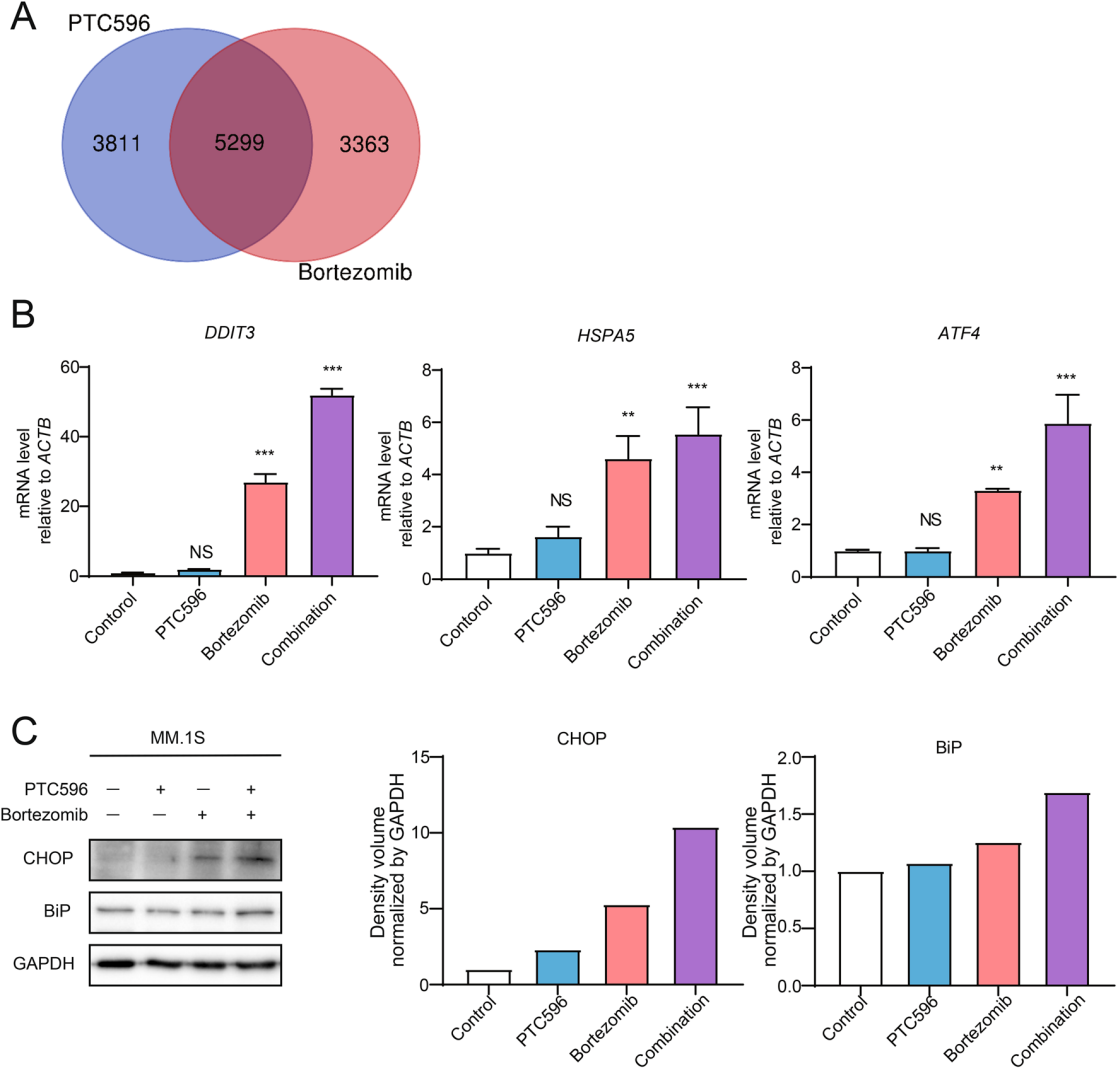
Endoplasmic reticulum stress is augmented by the combination of PTC596 and bortezomib. (**A**) Venn diagram of 9110 genes upregulated upon PTC596 treatment and 8662 genes upregulated upon bortezomib treatment, compared with the control, in RNA-seq analysis. Gene ontology terms related to endoplasmic reticulum were significantly enriched in 5299 overlapping genes. (**B**) Quantitative RT-PCR analysis of *BMI1* in MM.1S treated with or without PTC596 (100 nM) for 48 h in the presence or absence of bortezomib (2.5 nM) for 24 h. *ACTB* was used to normalize the amount of input RNA. Data are shown as mean ± SD (n = 3). **P < 0.01; ***P < 0.001; ns, not significant using one-way ANOVA. (**C**) Western blotting analysis of the indicated proteins in MM.1S. MM.1S were treated with or without PTC596 (100 nM) for 48 h in the presence or absence of bortezomib (2.5 nM) for 24 h. GAPDH served as a loading control. The right graphs show the density volume normalized by GAPDH.

In order to elucidate the target pathways of PTC596 and the mechanism underlying the synergistic effect with bortezomib, we analyzed RNA-seq data using g:profiler^26^. Genes up-regulated upon treatment with PTC596 or bortezomib as single agents largely overlapped. Of interest, endoplasmic reticulum (ER)-related gene ontology terms were significantly enriched in 5299 overlapping genes (Fig. 6A, Supplementary Table S7). RT-PCR analyses revealed the upregulation of representative ER stress-related genes, such as *DDIT3* (also known as CHOP or GADD153), *HSPA5* (also known as BiP or GRP78), and *ATF4*, by bortezomib treatment and to a lesser extent by PTC596 treatment. Of note, the expression of ER stress-related genes induced by bortezomib^27^ was significantly enhanced by the combination treatment (Fig. 6B). Among ER stress-related genes, *DDIT3* encodes a transcriptional factor CHOP which is related to fatal ER stress^27^. We confirmed that the protein levels of CHOP and BiP were elevated by the combination treatment by western blotting (Fig. 6C). Importantly, knockdown of *ATF4* and *DDIT3* by shRNA lead to the suppression of the cytotoxicity of the combination treatment, indicating that the ER stress pathway at least partially contributes to the synergy of PTC596 with bortezomib (Supplementary Fig. S4).

To look at the upregulation of ER stress pathways by the combination treatment in vivo*,* we performed western blotting and RT-PCR using tumors harvested from NOG mice treated with or without oral PTC596 in combination with or without subcutaneous bortezomib for 2 weeks. The protein levels of CHOP were enhanced by the combination treatment (N = 2, Supplementary Fig. S5A). We also confirmed that the mRNA expression of *DDIT3* and *ATF4* in the tumors was elevated by the combination treatment (Supplementary Fig. S5B). These data indicated that augmented ER stress is one of the mechanisms of action in this combination.

## Discussion

In this study, we demonstrated the activities of a novel tubulin binding agent PTC596 alone and in combination with proteasome inhibitors in MM both in vitro and in vivo. As reported recently in pancreatic ductal adenocarcinoma^11^, we confirmed that PTC596 inhibits tubulin polymerization in MM cells. PTC596-induced G2/M cell cycle arrest in MM cells may be attributed to the inhibitory effect of PTC596 against microtubule polymerization^28^. PTC596 significantly induced cytotoxicity against MM cell lines, primary MM cells, as well as MM cells in a xenograft model in vivo. Moreover, PTC596 induced significant cytotoxicity in MM cell lines regardless of bortezomib sensitivity or the status of *TP53* gene mutations and deletions, which are closely related to shorter survival, poor response, and resistance to drugs in MM^29^. These results indicated the sufficient preclinical efficacy of PTC596 in the treatment of MM, consistent with the recent report^15^.

As therapeutic strategies for MM, combination treatments including proteasome inhibitors or IMiDs are crucial to control this heterogeneous disease^30^. We found that combination treatment of PTC596 and proteasome inhibitors exerted additive or synergistic cytotoxicity in MM. Our in vivo data using a xenograft model demonstrated that this combination is promising, with definitive synergy and acceptable tolerability. Of note, fatal ER stress was identified as a possible mechanism of action in the combination of PTC596 with bortezomib. ER stress plays an important role in the development of MM and its pathway is a therapeutic target for proteasome inhibitors^31^. The ER stress pathway is also the target for the synergistic effects of combination treatments using various agents with bortezomib in MM^7,8,32,33^. We demonstrated that the transcription factor CHOP, a surrogate maker of fatal ER stress, was elevated in the combination of PTC596 with bortezomib compared with each single treatment. Although the mechanism by which PTC596 enhanced bortezomib-inducing ER stress is still unclear, a microtubule degrader N-deacetyl-N-(chromone-2-carbonyl)-thiocolchicine has been reported to induce ER stress-mediated cytotoxicity in hepatocellular carcinoma cells^34^. Podophyllotoxin acetate, which inhibits microtubule polymerization, also activates the ER stress pathway in non-small cell lung cancer cells^35^. These reports indicated that the microtubule disruption as well as inhibition of microtubule polymerization induce activation of the ER stress pathway.

MCL1 can be another target of PTC596 as a single agent as well as in combination with bortezomib. MCL1, an anti-apoptotic protein, is essential for MM cell survival and related to relapse and poor prognosis^36^. In the present study, PTC596 decreased MCL1 levels in MM cells, as reported previously in acute myeloid leukemia and mantle cell lymphoma^13,14^. Bortezomib treatment compromises the anti-apoptotic function of MCL1 by promoting its proteolytic cleavage^37,38^. Additive effects of PTC596 and bortezomib on the reduction of full length MCL1 protein may account for enhanced apoptosis with the combination therapy in MM cells.

As reported previously^13,14^, PTC596 reduced the levels of BMI1 and ubiquitination of H2A in MM cell lines. PTC596 reportedly induces the reduction in BMI1 via its phosphorylation at 2 N-terminal sites^12–14,39^; however, BMI1 becomes hyperphosphorylated and dissociates from chromatin during mitosis^11^, suggesting that PTC596 induces reductions in BMI1 protein levels as an indirect consequence of the induction of mitotic arrest. BMI1 plays an essential role in MM cell progression, survival, and drug resistance^40,41^. We confirmed that MM cells depend on BMI1 for their growth (Supplementary Fig. S6). However, unexpectedly, BMI1 target genes in MM cells were not significantly de-repressed by PTC596 treatment, suggesting that PTC596 functions independently of BMI1. Supporting our findings, a recent report has demonstrated that BMI1 is not required for the anti-MM activity of PTC596^15^. Nevertheless, bortezomib significantly downregulated BMI1 in MM cells as reported in mantle cell lymphoma cells^23^, and PTC596 enhanced the bortezomib-induced reductions in BMI1 protein levels in MM cells. Because depletion of *BMI1* was reported to enhance the sensitivity of MM cells to bortezomib^42^, the cooperative effects of PTC596 and bortezomib on BMI1 protein levels might be another mechanism of the synergistic action of this combination.

In conclusion, our findings demonstrate that microtubule polymerization inhibition alone and in combination with proteasome inhibition are potential novel therapeutic options in MM. This study provides a preclinical framework for the clinical evaluation of this promising therapeutic approach to improve outcomes in patients with MM.

## Materials and methods

### Reagents

PTC596 was developed and provided by PTC Therapeutics, South Plainfield, NJ, USA. It was diluted in DMSO to make 20 mM or 1 mM stocks for in vitro experiments. For in vivo experiments, it was diluted in 0.5% (w/v) hydroxypropyl methylcellulose solution with 0.1% (w/v) Tween 80 to make a 12.5 mg/mL stock. Bortezomib and carfilzomib for in vitro experiments was obtained from Selleck Chemicals and diluted in DMSO to make a 100 μM stock. Bortezomib for in vivo experiments was purchased from Janssen Pharmaceutical KK and was diluted in normal saline to make a 1 mg/mL stock. Paclitaxel was purchased from Sigma-Aldrich and was diluted in DMSO to make a 10 mM stock.

### Human MM cell lines and primary cells

Human MM cell lines MM.1S, NCI-H929 (H929), U266, and RPMI8226 were obtained from the American Type Culture Collection. Human KMS11 and bortezomib-resistant KMS11/BTZ^43^ cells were obtained from the Japanese Collection of Research Bioresources Cell Bank. Human OPM-2 plasma cell leukemia cell line was kindly provided by Dr. Edward Thompson (University of Texas Medical Branch, Galveston, TX). Bortezomib-resistant OPM-2/BTZ^43^ was obtained from Kyowa Kirin Co., Ltd. MM cells were cultured in RPMI 1640 containing 10% fetal bovine serum (FBS), 2 μM l-glutamine, 100 U/mL penicillin, and 100 μg/mL streptomycin (Thermo Fisher).

### Human samples from patients

Primary MM cells and BMSCs were collected from the bone marrow of patients with newly diagnosed or relapsed refractory MM at Chiba University Hospital. All patients provided written informed consent in accordance with the declaration of Helsinki and patient anonymity was ensured. This study was approved by the Institutional Review Committee at Chiba University (Approval #964 and #1025). Plasma cells and BMSCs were purified as previously described^9^ and were cultured in Dulbecco’s modified Eagle’s medium supplemented with 10% FBS, 2 μM l-glutamine, 100 U/mL penicillin, and 100 μg/mL streptomycin (Thermo Fisher).

### Co-culture experiments

BMSCs were plated and cultured in a 96 well plate for 24 h and then MM cells were added and treated with PTC596 in combination with bortezomib or carfilzomib for the indicated times. Then the samples were analyzed by assays of cytotoxicity.

### Assays of cytotoxicity

To evaluate the cytotoxicity of PTC596 alone and in combination with proteasome inhibitors, MTS and BrdU ELISA assays were performed using human MM cell lines. In MTS assay, CellTiter 96 AQueous One Solution (Promega) was added to the cells in the last 4 h of the incubation period and absorbance was read on a plate reader as described previously^9^. For BrdU ELISA assay, BrdU cell proliferation ELISA kits (colorimetric; Abcam and Roche) were used in accordance with the manufacturer’s instructions. The absorbance of BMSCs alone was subtracted from each value when MM cells were co-cultured with BMSCs.

### Murine xenograft models of human MM

Male NOD/Shi-scid, IL-2RγKOJic (NOG) mice were purchased from CLEA Japan Inc. Animal studies of xenograft models were conducted according to Chiba University guidelines for the use of laboratory animals and approved by the Review Board for Animal Experiments of Chiba University (approval #1–92).

NOG mice were injected subcutaneously on the right side of the back with 4 × 10^6^ MM.1S cells in 100 μL RPMI 1640. After the tumor grew to a measurable size, treatment was initiated. For the PTC596 single treatment described in Fig. 1D and E, the mice were treated with oral PTC596 (12.5 mg/kg) or control vehicle twice a week for 4 weeks. For the combination treatment of PTC596 with bortezomib, the mice were treated with oral PTC596 (6.25 mg/kg) and subcutaneous saline twice a week; subcutaneous bortezomib (0.5 mg/kg) in the left side of the back and orally administered vehicle twice a week; or oral PTC596 (6.25 mg/kg) twice a week and subcutaneous bortezomib (0.5 mg/kg) twice a week. Control mice received orally administered vehicle and subcutaneous saline. For the evaluation of tumor volume and survival analysis described in Fig. 4A–C, the mice were treated for 5 weeks. Tumor volume was calculated from caliper measurements every 3–4 days until the day of the first death in each group. The mice were sacrificed when the tumors reached 2,000 cm^3^ or an ulcer occurred. Survival was evaluated from the first day of treatment to death. Hematological toxicity described in Fig. 4D,E and the ER-stress induction described in Supplementary Fig. S5 were examined in another cohort, in which the mice were treated with or without oral PTC596 (6.25 mg/kg) twice a week in combination with or without subcutaneous bortezomib (0.5 mg/kg) twice a week for 2 weeks.

### Western blotting

MM cells were lysed and sonicated prior to SDS-PAGE as described previously^9^. Tumors harvested from NOG mice were homogenized using a TissueRuptor (Qiagen) and lysed in RIPA (50 mM Tris, pH 8.0, 150 mM NaCl,1 mM EDTA, pH 8.0, 1% TritonX-100, 0.1% sodium deoxycholate and 0.1% SDS) buffers with protease inhibitor cocktail (Roche). Then Lysates were sonicated using a Bioruptor (COSMO BIO CO.) The supernatants were separated after centrifugation and mixed with SDS-sample buffer (25 mM Tris, pH 6.8, 1% SDS, 5% glycerol, 0.05% bromophenol blue and 1%-mercaptoethanol).

Immunoblotting was performed using the following antibodies: anti-BMI1(Bethyl), anti-uH2A, anti-H2A, anti-caspase-3, anti-caspase-8, anti-caspase-9, anti-PARP, anti-CHOP, anti-BiP, and anti-GAPDH (Cell Signaling), anti-MCL1 (Santa Cruz) and anti-α-tubulin (Calbiochem). Whole blots are available in the Supplementary Information file.

### Immunoblotting analysis of soluble versus polymerized tubulin

MM.1S cells were treated with PTC596 or paclitaxel for 4 h. As reported previously^44^, after washing by PBS, the cells were permeabilized with 200 μL of pre-warmed buffer [80 mM PIPES-KOH, 2 mM MgCl_2_, 0.5 mM EGTA, 0.2% Triton X-100, 10% glycerol, 1 × Protease inhibitor, pH 6.9] and incubated for 5 min at 30 °C. The supernatants containing the soluble fraction of microtubules were separated after centrifugation and mixed with 4 × Laemmli gel sample buffer. To collect the insoluble (polymerized) tubulin fraction, 1 × Laemmli gel sample buffer (250 μL) was added to the pellet. These samples were boiled for 3 min. Microtubules were detected by western blotting with anti-α-tubulin antibodies.

### Analysis of cell cycle and apoptosis by flow cytometry

An FITC BrdU Flow kit (BD Pharmigen) was used for cell cycle analysis in MM cells, and an FITC Annexin V Apoptosis Detection Kit I (BD Pharmingen) was used for the detection of apoptotic MM cells, in accordance with the manufacturer’s instructions. Flow cytometry was performed using a BD FACS Canto II (BD Biosciences), and the obtained data were analyzed using FlowJo software (Tree Star).

### RNA sequencing

Total RNA was isolated from ~ 1.0 × 10^6^ MM.1S or OPM-2 cells using an RNeasy Mini Kit (QIAGEN). RNA concentration and integrity were verified using an Agilent 2100 Bioanalyzer (Santa Clara, CA, USA). Amplification, preparation of the libraries, and RNA sequencing (RNA-seq) were performed as described previously^45,46^.

TopHat (version 1.3.2; with default parameters) was used to align with the human reference genome (hg19 from University of California, Santa Cruz Genome Browser; http://genome.ucsc.edu/). Then, gene expression values were calculated as reads per kilobase of exon unit per million mapped reads (RPKM) using cufflinks (version 2.0.2).

### Quantitative RT-PCR

Total RNA was purified using an RNeasy Mini Kit or RNeasy plus micro kit (QIAGEN). cDNA was made using the ThermoScript RT-PCR system (Invitrogen) or a PrimeScript™ RT reagent Kit (Perfect Real Time) (Takara) with an oligo-dT primer. Quantitative RT-PCR was performed on a StepOnePlus Real-Time PCR System (Thermo Fisher Scientific) by using TB Green™ Premix Ex Taq™ GC (Perfect Real Time) (Takara). All data were examined in triplicate and presented as relative expression levels normalized to *GAPDH* or *ACTB* (β-actin) expression. The sequences of forward and reverse primers are shown in Supplementary Table S8.

### Vector and virus production

A retroviral vector and virus were made using the same method as described previously^47^. *Mouse Bmi1 (mBmi1)* cDNA tagged with a 3 × Flag in the retroviral vector pGCDNsam was used for the overexpression of mBmi1 to perform mBmi1 chromatin immunoprecipitation sequencing (ChIP-seq). For *BMI1* knockdown experiments, lentiviral vectors expressing short hairpin RNA (shRNA) that target human *BMI1* (sh-*BMI1*) and *luciferase* (sh-*Luc*) were produced as described previously^48^. Knockdown experiments of *DDIT3* and *ATF4* were performed using lentiviral vectors: pLV[shRNA]-EGFP:T2A:Puro-U6 > hATF4[sh-*ATF4#1*], pLV[shRNA]-EGFP:T2A:Puro-U6 > hATF4[sh-*ATF4#2*], pLV[shRNA]-EGFP:T2A:Puro-U6 > hDDIT3[sh-*DDIT3#1*], pLV[shRNA]-EGFP:T2A:Puro-U6 > hDDIT3[sh-*DDIT3#2*] and pLV[shRNA]-EGFP/Puro-U6 > Scramble_shRNA[sh-Scramble], constructed by VectorBuilder. Short hairpin sequences used in this study are sh-*DDIT3#1*: CTGCACCAAGCATGAACAATT, sh-*DDIT3#2*: TGAACGGCTCAAGCAGGAAAT, sh-*ATF4#1*: CATGATCCCTCAGTGCATAAA, sh-*ATF4#2*: TGAACGGCTCAAGCAGGAAAT. Recombinant retroviruses and lentiviruses were produced using established methods^49^.

### Chromatin immunoprecipitation sequencing

mBmi1 ChIP-seq was performed in mBmi1-overexpressing MM.1S cells as described previously^50^. Immunoprecipitation was performed using an anti-FLAG antibody (Sigma). Sheep anti-mouse IgG Dynabeads were used to capture the antibody.

Bowtie2 (version 2.2.6; default parameters) was used to map the reads to the reference genome (UCSC/mm10). Peaks were called using MACS2 v2.1.1 with a q-value of < 0.1 for mBMI1. ChIP peaks that overlapped with those of a corresponding input (distance between centers < 10 kb) were removed. Reads per million mapped reads (RPM) values of the sequenced reads were calculated for every 1000 bp bin, with a shifting size of 100 bp using Bedtools. To visualize with Integrative GenomicsViewer (Broad Institute), the RPM values of the immunoprecipitated samples were normalized by subtracting the RPM values of the input samples in each bin and converted to a Bigwig file using the Wigtobigwig tool. In order to evaluate the mark of each gene, the RPM values of the region from 2 kb upstream to 2 kb downstream of the TSS of immunoprecipitated samples were divided by the RPM of the corresponding input.

### Statistical analysis

The statistical significance of differences was measured using an unpaired two-tailed Student's *t*-test or Welch’s *t-*test when the variance was judged as significantly different.

For multiple comparisons of combination treatments, one-way ANOVA test and Tukey’s test were performed. P values less than 0.05 were considered significant, using Graph Pad Prism, version 8. Survival analysis by Kaplan–Meier curves and log-rank analysis were performed using Graph Pad Prism, version 8. The combination index (CI) of PTC596 with bortezomib was analyzed by isobologram analysis using Compu-Syn software (ComboSyn, Inc.)^19^. CI values of less than 1.0, equal to 1.0, and greater than 1.0 indicate synergistic, additive, and antagonistic effects, respectively.

### Deposition of data

RNA sequence data were deposited in the DNA Data Bank of Japan (DDBJ) (accession number: DRA009600).

## Supplementary Information

Supplementary Information.
