# Supplementary material for: The combination of the tubulin binding small molecule PTC596 and proteasome inhibitors suppresses the growth of myeloma cells
Source: Sci Rep. 2021 Jan 22;11:2074. doi: 10.1038/s41598-021-81577-x (PMC7822878; doi:10.1038/s41598-021-81577-x)

The combination of the tubulin binding small molecule PTC596 and proteasome inhibitors suppresses the growth of myeloma cells

Yurie Nagai, Naoya Mimura, Ola Rizq, Yusuke Isshiki, Motohiko Oshima, Mohamed Rizk, Atsunori Saraya, Shuhei Koide, Yaeko Nakajima-Takagi, Makiko Miyota, Tetsuhiro Chiba, Nagisa Oshima-Hasegawa, Tomoya Muto, Shokichi Tsukamoto, Shio Mitsukawa, Yusuke Takeda, Chikako Ohwada, Masahiro Takeuchi, Tohru Iseki, Chiaki Nakaseko, William Lennox, Josephine Sheedy, Marla Weetall, Koutaro Yokote, Atsushi Iwama, and Emiko Sakaida

<u>Supporting Information:</u>

Supplementary Tables S1-S8

Supplementary Figures and legends S1-S6

Whole blots for cropped images

Supplementary Table S1.

CC_{50} values of cell lines calculated from the results of MTS assays described in Figure 1A, B.

	MM.1S	H929	U266	RPMI 8226	KMS-11	KMS-11 /BTZ	OPM-2	OPM-2 /BTZ
CC50 [nM]	45.58	44.03	24.19	50.75	49.46	48.33	97.74	74.21

Supplementary Table S2.

The combination effect of two agents was analyzed by isobologram analysis with Compu-Syn software (ComboSyn, Inc).

(A) CI values of MM.1S cells treated with the combination of PTC596 and bortezomib in a BrdU proliferation assay (Figure 3A).

(B) CI values of MM.1S cells treated with the combination of PTC596 and bortezomib in an MTS assay (Figure S1).

(C) CI values of OPM-2 cells treated with the combination of PTC596 and bortezomib in a BrdU proliferation assay (Figure S2).

(D) CI values of MM.1S cells treated with the combination of PTC596 and carfilzomib in a BrdU proliferation assay (Figure S3).

Supplementary Table S2A

PTC596 [nM]	Bortezomib [nM]	Fa	CI
60.0	2.0	0.4031	0.88409
60.0	2.5	0.5842	0.70830
60.0	3.0	0.6315	0.75578
90.0	2.0	0.4296	0.82729
90.0	2.5	0.5813	0.71339
90.0	3.0	0.6719	0.68030

Supplementary Table S2B

PTC596 [nM]	Bortezomib [nM]	Fa	CI
20.0	4.0	0.1804	1.28328
20.0	5.0	0.5075	1.12710
40.0	4.0	0.2120	1.50260
40.0	5.0	0.6441	1.12023
60.0	4.0	0.4290	1.30784
60.0	5.0	0.5819	1.29851
80.0	4.0	0.5004	1.34579
80.0	5.0	0.6145	1.36450

Supplementary Table S2C

PTC596 [nM]	Bortezomib [nM]	Fa	CI
60.0	4.0	0.6469	1.01406
60.0	5.0	0.8250	1.06931
125.0	4.0	0.8491	0.88074
125.0	5.0	0.8385	1.09679
250.0	4.0	0.7795	1.07512
250.0	5.0	0.8790	1.09907

Supplementary Table S2D

PTC596 [nM]	Carfilzomib [nM]	Fa	CI
15	3	0.4271	1.20322
15	4	0.7983	0.86478
15	5	0.9473	0.77541
30	3	0.8492	0.63050
30	4	0.9374	0.65286
30	5	0.9562	0.75073

Supplementary Table S3.

Significantly upregulated gene sets in MM.1S cells treated with PTC596.

Gene set enrichment analysis using our RNA-seq data identified gene sets, which were significantly enriched in MM.1S cells treated with PTC596 versus control. (FDR w-value <0.01).

c2.all.v7.0

NAME	SIZE	NES	NOM p-val	FDR q-val
MACAEVA_PBMC_RESPONSE_TO_IR	96	2.42	0.000	0.000
KERLEY_RESPONSE_TO_CISPLATIN_UP	43	2.33	0.000	0.001

Supplementary Table S4.

Significantly downregulated gene sets in MM.1S cells treated with PTC596.

Gene set enrichment analysis using our RNA-seq data identified gene sets, which were significantly downregulated in MM.1S cells treated with PTC596 versus control. (FDR w-value <0.01).

Hallmark

NAME	SIZE	NES	NOM p-val	FDR q-val
HALLMARK_E2F_TARGETS	196	-3.03	0.000	0.000
HALLMARK_G2M_CHECKPOINT	189	-2.42	0.000	0.000
HALLMARK_MYC_TARGETS_V1	197	-2.34	0.000	0.000
HALLMARK_ESTROGEN_RESPONSE_LATE	164	-2.00	0.000	0.002
HALLMARK_DNA_REPAIR	146	-1.87	0.001	0.005

c2.all.v7.0

NAME	SIZE	NES	NOM p-val	FDR q-val
DUTERTRE_ESTRADIOL_RESPONSE_24HR_UP	303	-3.23	0.000	0.000
KOBAYASHI_EGFR_SIGNALING_24HR_DN	244	-3.08	0.000	0.000
ROSTY_CERVICAL_CANCER_PROLIFERATION_CLUSTER	135	-2.96	0.000	0.000
FUJII_YBX1_TARGETS_DN	187	-2.95	0.000	0.000
BURTON_ADIPOGENESIS_3	96	-2.93	0.000	0.000
VERNELL_RETINOBLASTOMA_PATHWAY_UP	69	-2.91	0.000	0.000
PUJANA_XPRSS_INT_NETWORK	161	-2.89	0.000	0.000
BLUM_RESPONSE_TO_SALIRASIB_DN	320	-2.89	0.000	0.000
GRAHAM_NORMAL_QUIESCENT_VS_NORMAL_DIVIDING_DN	85	-2.88	0.000	0.000
RUIZ_TNC_TARGETS_DN	140	-2.87	0.000	0.000
WHITEFORD_PEDIATRIC_CANCER_MARKERS	111	-2.86	0.000	0.000
ZHOU_CELL_CYCLE_GENES_IN_IR_RESPONSE_6HR	79	-2.86	0.000	0.000
PUJANA_BRCA2_PCC_NETWORK	403	-2.84	0.000	0.000
SHEDDEN_LUNG_CANCER_POOR_SURVIVAL_A6	428	-2.83	0.000	0.000
MISSIAGLIA_REGULATED_BY_METHYLATION_DN	115	-2.82	0.000	0.000
WHITFIELD_CELL_CYCLE_G1_S	125	-2.81	0.000	0.000
PUJANA_BRCA_CENTERED_NETWORK	116	-2.81	0.000	0.000
REACTOME_DNA_REPLICATION	123	-2.79	0.000	0.000
SOTIRIOU_BREAST_CANCER_GRADE_1_VS_3_UP	147	-2.79	0.000	0.000
SARRIO_EPITHELIAL_MESENCHYMAL_TRANSITION_UP	175	-2.78	0.000	0.000
CROONQUIST_IL6_DEPRIVATION_DN	93	-2.78	0.000	0.000
ZHOU_CELL_CYCLE_GENES_IN_IR_RESPONSE_24HR	116	-2.77	0.000	0.000
LEE_EARLY_T_LYMPHOCYTE_UP	99	-2.77	0.000	0.000
FISCHER_G1_S_CELL_CYCLE	188	-2.76	0.000	0.000
WINNEPENNINCKX_MELANOMA_METASTASIS_UP	156	-2.76	0.000	0.000
GRAHAM_CML_DIVIDING_VS_NORMAL_QUIESCENT_UP	169	-2.76	0.000	0.000

WANG_RESPONSE_TO_GSK3_INHIBITOR_SB216763_DN	336	-2.75	0.000	0.000
KONG_E2F3_TARGETS	91	-2.75	0.000	0.000
CHICAS_RB1_TARGETS_SENESCENT	488	-2.73	0.000	0.000
CROONQUIST_NRAS_SIGNALING_DN	69	-2.71	0.000	0.000
SONG_TARGETS_OF_IE86_CMV_PROTEIN	54	-2.70	0.000	0.000
KAUFFMANN_MELANOMA_RELAPSE_UP	61	-2.69	0.000	0.000
BENPORATH_PROLIFERATION	140	-2.69	0.000	0.000
REACTOME_DNA_REPLICATION_PRE_INITIATION	82	-2.68	0.000	0.000
REACTOME_DNA_STRAND_ELONGATION	32	-2.68	0.000	0.000
MANALO_HYPOXIA_DN	283	-2.68	0.000	0.000
REACTOME_MITOTIC_G1_G1_S_PHASES	145	-2.67	0.000	0.000
FLORIO_NEOCORTEX_BASAL_RADIAL_GLIA_DN	166	-2.66	0.000	0.000
REACTOME_S_PHASE	156	-2.66	0.000	0.000
REACTOME_ACTIVATION_OF_ATR_IN_RESPONSE_TO_REPLICATION_STRESS	37	-2.66	0.000	0.000
MARKEY_RB1_ACUTE_LOF_UP	214	-2.65	0.000	0.000
CHICAS_RB1_TARGETS_GROWING	214	-2.65	0.000	0.000
CHIANG_LIVER_CANCER_SUBCLASS_PROLIFERATION_UP	163	-2.64	0.000	0.000
STEIN_ESRRA_TARGETS_RESPONSIVE_TO_ESTROGEN_DN	39	-2.63	0.000	0.000
BILD_E2F3_ONCOGENIC_SIGNATURE	223	-2.63	0.000	0.000
KEGG_DNA_REPLICATION	36	-2.62	0.000	0.000
BERENJENO_TRANSFORMED_BY_RHOA_UP	497	-2.62	0.000	0.000
KAUFFMANN_DNA_REPLICATION_GENES	139	-2.62	0.000	0.000
HOFFMANN_LARGE_TO_SMALL_PRE_BII_LYMPHOCYTE_UP	142	-2.61	0.000	0.000
LINDGREN_BLADDER_CANCER_CLUSTER_3_UP	306	-2.61	0.000	0.000
REACTOME_HDR_THROUGH_HOMOLOGOUS_RECOMBINATION_HRR	64	-2.61	0.000	0.000
MORI_LARGE_PRE_BII_LYMPHOCYTE_UP	81	-2.60	0.000	0.000
LE_EGR2_TARGETS_UP	101	-2.60	0.000	0.000
TOYOTA_TARGETS_OF_MIR34B_AND_MIR34C	417	-2.60	0.000	0.000
REACTOME_ACTIVATION_OF_THE_PRE_REPLICATIVE_COMPLEX	33	-2.59	0.000	0.000
ISHIDA_E2F_TARGETS	50	-2.59	0.000	0.000
MORI_IMMATURE_B_LYMPHOCYTE_DN	86	-2.59	0.000	0.000
KANG_DOXORUBICIN_RESISTANCE_UP	52	-2.59	0.000	0.000
VECCHI_GASTRIC_CANCER_EARLY_UP	388	-2.59	0.000	0.000
WONG_EMBRYONIC_STEM_CELL_CORE	325	-2.58	0.000	0.000
DUTERTRE_ESTRADIOL_RESPONSE_6HR_UP	214	-2.58	0.000	0.000
PYEON_HPV_POSITIVE_TUMORS_UP	86	-2.58	0.000	0.000
REACTOME_HOMOLOGOUS_DNA_PAIRING_AND_STRAND_EXCHANGE	41	-2.57	0.000	0.000
PYEON_CANCER_HEAD_AND_NECK_VS_CERVICAL_UP	177	-2.56	0.000	0.000
LI_WILMS_TUMOR_VS_FETAL_KIDNEY_1_DN	155	-2.56	0.000	0.000
MITSIADES_RESPONSE_TO_APLIDIN_DN	241	-2.55	0.000	0.000
MOLENAAR_TARGETS_OF_CCND1_AND_CDK4_DN	52	-2.55	0.000	0.000
REACTOME_EXTENSION_OF_TELOMERES	30	-2.55	0.000	0.000

MUELLER_PLURINET	286	-2.54	0.000	0.000
WHITFIELD_CELL_CYCLE_S	143	-2.52	0.000	0.000
PUJANA_BREAST_CANCER_WITH_BRCA1_MUTATED_UP	54	-2.52	0.000	0.000
SENGUPTA_NASOPHARYNGEAL_CARCINOMA_UP	276	-2.52	0.000	0.000
GARCIA_TARGETS_OF_FLI1_AND_DAX1_DN	150	-2.52	0.000	0.000
REN_BOUND_BY_E2F	60	-2.51	0.000	0.000
FOURNIER_ACINAR_DEVELOPMENT_LATE_2	273	-2.51	0.000	0.000
STEIN_ESR1_TARGETS	73	-2.51	0.000	0.000
PID_FANCONI_PATHWAY	45	-2.50	0.000	0.000
BURTON_ADIPOGENESIS_PEAK_AT_16HR	38	-2.50	0.000	0.000
REACTOME_MITOTIC_PROMETAPHASE	191	-2.50	0.000	0.000
FRASOR_RESPONSE_TO_SERM_OR_FULVESTRANT_DN	45	-2.50	0.000	0.000
AFFAR_YY1_TARGETS_DN	197	-2.50	0.000	0.000
REACTOME_HDR_THROUGH_SINGLE_STRAND_ANNEALING_SSA	36	-2.50	0.000	0.000
ZHENG_GLIOBLASTOMA_PLASTICITY_UP	209	-2.49	0.000	0.000
KAUFFMANN_DNA_REPAIR_GENES	225	-2.45	0.000	0.000
PID_ATR_PATHWAY	39	-2.45	0.000	0.000
KAMMINGA_EZH2_TARGETS	41	-2.44	0.000	0.000
ZHAN_MULTIPLE_MYELOMA_PR_UP	43	-2.43	0.000	0.000
REACTOME_G1_S_SPECIFIC_TRANSCRIPTION	28	-2.43	0.000	0.000
REACTOME_TELOMERE_C_STRAND_LAGGING_STRAND_SYNTHESIS	24	-2.42	0.000	0.000
MARKEY_RB1_CHRONIC_LOF_UP	98	-2.41	0.000	0.000
BASAKI_YBX1_TARGETS_UP	268	-2.41	0.000	0.000
BENPORATH_ES_1	341	-2.41	0.000	0.000
MORI_PRE_BI_LYMPHOCYTE_UP	74	-2.41	0.000	0.000
VANTVEER_BREAST_CANCER_METASTASIS_DN	119	-2.41	0.000	0.000
RHEIN_ALL_GLUCOCORTICOID_THERAPY_DN	350	-2.40	0.000	0.000
EGUCHI_CELL_CYCLE_RB1_TARGETS	23	-2.39	0.000	0.000
ODONNELL_TARGETS_OF_MYC_AND_TFRC_DN	43	-2.39	0.000	0.000
RHODES_UNDIFFERENTIATED_CANCER	66	-2.39	0.000	0.000
REACTOME_CELL_CYCLE_CHECKPOINTS	267	-2.38	0.000	0.000
REACTOME_RECOGNITION_OF_DNA_DAMAGE_BY_PCNA_CONTAINING_REPLICATION_COMPLEX	30	-2.38	0.000	0.000
FURUKAWA_DUSP6_TARGETS_PCI35_DN	63	-2.37	0.000	0.000
FERRANDO_T_ALL_WITH_MLL_ENL_FUSION_DN	77	-2.37	0.000	0.000
REACTOME_DNA_DAMAGE_BYPASS	45	-2.36	0.000	0.000
REACTOME_RESOLUTION_OF_ABASIC_SITES_AP_SITES	38	-2.36	0.000	0.000
MORI_EMU_MYC_LYMPHOMA_BY_ONSET_TIME_UP	102	-2.36	0.000	0.000
ODONNELL_TFRC_TARGETS_DN	120	-2.35	0.000	0.000
VILLANUEVA_LIVER_CANCER_KRT19_UP	164	-2.35	0.000	0.000
REACTOME_RESOLUTION_OF_SISTER_CHROMATID_COHESION	118	-2.35	0.000	0.000
PID_E2F_PATHWAY	68	-2.35	0.000	0.000

RIZ_ERYTHROID_DIFFERENTIATION	78	-2.35	0.000	0.000
WHITFIELD_CELL_CYCLE_LITERATURE	41	-2.34	0.000	0.000
HORIUCHI_WTAP_TARGETS_DN	294	-2.34	0.000	0.000
REACTOME_ASSEMBLY_OF_THE_PRE_REPLICATIVE_COMPLEX	65	-2.34	0.000	0.000
REACTOME_GAP_FILLING_DNA_REPAIR_SYNTHESIS_AND_LIGATION_IN_GG_NER	25	-2.34	0.000	0.000
FERREIRA_EWINGS_SARCOMA_UNSTABLE_VS_STABLE_UP	148	-2.34	0.000	0.000
GOLDRATH_ANTIGEN_RESPONSE	316	-2.32	0.000	0.000
REACTOME_RESOLUTION_OF_AP_SITES_VIA_THE_MULTIPLE_NUCLEOTIDE_PATCH_REPLACEMENT_PATHWAY	25	-2.32	0.000	0.000
PUJANA_BREAST_CANCER_LIT_INT_NETWORK	98	-2.31	0.000	0.000
REACTOME_PCNA_DEPENDENT_LONG_PATCH_BASE_EXCISION_REPAIR	21	-2.31	0.000	0.000
SUNG_METASTASIS_STROMA_DN	47	-2.30	0.000	0.000
REACTOME_LAGGING_STRAND_SYNTHESIS	20	-2.30	0.000	0.000

Supplementary Table S5.

Significantly upregulated gene sets in MM.1S cells treated with combination treatment of PTC596 with bortezomib.

Gene set enrichment analysis using our RNA-seq data identified gene sets, which were significantly enriched in MM.1S cells treated with the combination treatment versus control. (FDR w-value <0.01).

Hallmark

NAME	SIZE	NES	NOM p-val	FDR q-val
HALLMARK_TNFA_SIGNALING_VIA_NFKB	183	2.31	0.000	0.000
HALLMARK_APOPTOSIS	152	2.12	0.000	0.000
HALLMARK_P53_PATHWAY	187	1.98	0.000	0.000
HALLMARK_HYPOXIA	182	1.88	0.000	0.000
HALLMARK_UV_RESPONSE_UP	148	1.71	0.000	0.006
HALLMARK_IL6_JAK_STAT3_SIGNALING	68	1.64	0.000	0.008
HALLMARK_REACTIVE_OXYGEN_SPECIES_PATHWAY	46	1.63	0.002	0.007
HALLMARK_UNFOLDED_PROTEIN_RESPONSE	109	1.60	0.000	0.008
HALLMARK_HEME_METABOLISM	176	1.60	0.000	0.007

c2.all.v7.0

NAME	SIZE	NES	NOM p-val	FDR q-val
PODAR_RESPONSE_TO_ADAPHOSTIN_UP	144	3.01	0.000	0.000
CONCANNON_APOPTOSIS_BY_EPOXOMICIN_UP	224	2.93	0.000	0.000
GARGALOVIC_RESPONSE_TO_OXIDIZED_PHOSPHOLIPIDS_BLUE_UP	128	2.70	0.000	0.000
REACTOME_HSP90_CHAPERONE_CYCLE_FOR_STEROID_HORMONE_RECEPTORS_SHR	54	2.52	0.000	0.000
REACTOME_THE_ROLE_OF_GTSE1_IN_G2_M_PROGRESSION_AFTER_G2_CHECKPOINT	74	2.51	0.000	0.000
REACTOME_AUF1_HNRNP_D0_BINDS_AND_DESTABILIZES_MRNA	52	2.49	0.000	0.000
REACTOME_DECTIN_1_MEDIATED_NONCANONICAL_NF_KB_SIGNALING	58	2.45	0.000	0.000
REACTOME_STABILIZATION_OF_P53	53	2.44	0.000	0.000
REACTOME_HSF1_DEPENDENT_TRANSACTIVATION	35	2.42	0.000	0.000
REACTOME_HEDGEHOG_LIGAND_BIOGENESIS	59	2.41	0.000	0.000
PICCALUGA_ANGIOIMMUNOBLASTIC_LYMPHOMA_DN	130	2.40	0.000	0.000
REACTOME_VIF_MEDIATED_DEGRADATION_OF_APOBEC3G	49	2.40	0.000	0.000
REACTOME_DEGRADATION_OF_AXIN	52	2.40	0.000	0.000
REACTOME_ABC_TRANSPORTER_DISORDERS	71	2.40	0.000	0.000
REACTOME_FCERI_MEDIATED_NF_KB_ACTIVATION	78	2.39	0.000	0.000
REACTOME_NEGATIVE_REGULATION_OF_NOTCH4_SIGNALING	53	2.38	0.000	0.000
KEGG_PROTEASOME	41	2.37	0.000	0.000
REACTOME_DEGRADATION_OF_GLI1_BY_THE_PROTEASOME	57	2.36	0.000	0.000
REACTOME_ACTIVATION_OF_NF_KAPPAB_IN_B_CELLS	64	2.36	0.000	0.000
REACTOME_CROSS_PRESENTATION_OF_SOLUBLE_EXOGENOUS_ANTIGENS_ENDOSOMES	43	2.36	0.000	0.000

REACTOME_MAPK6_MAPK4_SIGNALING	83	2.35	0.000	0.000
REACTOME_FBXL7_DOWN_REGULATES_AURKA_DURING_MITOTIC_ENTRY_AND_IN_EARLY_MITOSIS	52	2.35	0.000	0.000
REACTOME_REGULATION_OF_PTEN_STABILITY_AND_ACTIVITY	65	2.35	0.000	0.000
REACTOME_REGULATION_OF_APOPTOSIS	50	2.35	0.000	0.000
REACTOME_CELLULAR_RESPONSE_TO_HYPOXIA	68	2.35	0.000	0.000
REACTOME_ATTENUATION_PHASE	26	2.35	0.000	0.000
GARGALOVIC_RESPONSE_TO_OXIDIZED_PHOSPHOLIPIDS_TURQUOISE_UP	76	2.34	0.000	0.000
REACTOME_INTERLEUKIN_1_SIGNALING	95	2.34	0.000	0.000
REACTOME_DEGRADATION_OF_DVL	54	2.33	0.000	0.000
REACTOME_ABC_FAMILY_PROTEINS_MEDIATED_TRANSPORT	95	2.33	0.000	0.000
REACTOME_DEFECTIVE_CFTR_CAUSES_CYSTIC_FIBROSIS	57	2.33	0.000	0.000
REACTOME_CLEC7A_DECTIN_1_SIGNALING	95	2.33	0.000	0.000
REACTOME_REGULATION_OF_MRNA_STABILITY_BY_ PROTEINS_THAT_BIND_AU_RICH_ELEMENTS	84	2.32	0.000	0.000
REACTOME_REGULATION_OF_RUNX3_EXPRESSION_AND_ACTIVITY	54	2.31	0.000	0.000
NOJIMA_SFRP2_TARGETS_UP	30	2.30	0.000	0.000
REACTOME_ANTIGEN_PROCESSING_CROSS_PRESENTATION	92	2.29	0.000	0.000
REACTOME_METABOLISM_OF_POLYAMINES	56	2.29	0.000	0.000
REACTOME_HSF1_ACTIVATION	29	2.28	0.000	0.000
REACTOME_TNFR2_NON_CANONICAL_NF_KB_PATHWAY	89	2.27	0.000	0.000
REACTOME_REGULATION_OF_RUNX2_EXPRESSION_AND_ACTIVITY	68	2.26	0.000	0.000
GERY_CEBP_TARGETS	104	2.25	0.000	0.000
BLUM_RESPONSE_TO_SALIRASIB_UP	235	2.24	0.000	0.000
REACTOME_DEGRADATION_OF_BETA_CATENIN_BY_THE_DESTRUCTION_COMPLEX	79	2.24	0.000	0.000
REACTOME_DOWNSTREAM_SIGNALING_EVENTS_OF_B_CELL_RECEPTOR_BCR	78	2.22	0.000	0.000
GARGALOVIC_RESPONSE_TO_OXIDIZED_PHOSPHOLIPIDS_MAGENTA_UP	27	2.22	0.000	0.000
REACTOME_G1_S_DNA_DAMAGE_CHECKPOINTS	63	2.21	0.000	0.000
REACTOME_HEDGEHOG_OFF_STATE	105	2.21	0.000	0.000
REACTOME_COPI_INDEPENDENT_GOLGI_TO_ER_RETROGRADE_TRAFFIC	50	2.21	0.000	0.000
KRIGE_AMINO_ACID_DEPRIVATION	27	2.21	0.000	0.000
HELLER_SILENCED_BY_METHYLATION_DN	102	2.20	0.000	0.000
REACTOME_REGULATION_OF_EXPRESSION_OF_SLITS_AND_ROBOS	158	2.20	0.000	0.000
LEONARD_HYPOXIA	42	2.20	0.000	0.000
TIEN_INTESTINE_PROBIOTICS_24HR_DN	201	2.19	0.000	0.000
REACTOME_SIGNALING_BY_NOTCH4	81	2.19	0.000	0.000
REACTOME_HEDGEHOG_ON_STATE	80	2.18	0.000	0.000
GALINDO_IMMUNE_RESPONSE_TO_ENTEROTOXIN	71	2.18	0.000	0.000
REACTOME_CELLULAR_RESPONSE_TO_HEAT_STRESS	96	2.18	0.000	0.000
BURTON_ADIPOGENESIS_PEAK_AT_2HR	39	2.17	0.000	0.000
REACTOME_C_TYPE_LECTIN_RECEPTORS_CLRS	124	2.16	0.000	0.000
KAN_RESPONSE_TO_ARSENIC_TRIOXIDE	99	2.16	0.000	0.000

REACTOME_UCH_PROTEINASES	96	2.15	0.000	0.000
CHEN_LVAD_SUPPORT_OF_FAILING_HEART_UP	82	2.15	0.000	0.000
REACTOME_DOWNSTREAM_TCR_SIGNALING	88	2.15	0.000	0.000
MITSIADES_RESPONSE_TO_APLIDIN_UP	423	2.15	0.000	0.000
NAGASHIMA_NRG1_SIGNALING_UP	157	2.14	0.000	0.000
REACTOME_SIGNALING_BY_HEDGEHOG	138	2.14	0.000	0.000
CHEN_HOXA5_TARGETS_9HR_UP	210	2.13	0.000	0.000
REACTOME_TRANSCRIPTIONAL_REGULATION_BY_RUNX3	93	2.13	0.000	0.000
REACTOME_ASYMMETRIC_LOCALIZATION_OF_PCP_PROTEINS	61	2.13	0.000	0.000
GROSS_HYPOXIA_VIA_ELK3_DN	133	2.13	0.000	0.000
CHO_NR4A1_TARGETS	24	2.13	0.000	0.000
CHUANG_OXIDATIVE_STRESS_RESPONSE_UP	26	2.12	0.000	0.000
REACTOME_EUKARYOTIC_TRANSLATION_INITIATION	114	2.12	0.000	0.000
DANG_MYC_TARGETS_DN	29	2.12	0.000	0.000
REACTOME_SCF_SKP2_MEDIATED_DEGRADATION_OF_P27_21	58	2.11	0.000	0.000
HOUSTIS_ROS	31	2.11	0.000	0.000
REACTOME_REGULATION_OF_RAS_BY_GAPS	65	2.11	0.000	0.000
AMUNDSON_RESPONSE_TO_ARSENITE	205	2.11	0.000	0.000
DALESSIO_TSA_RESPONSE	18	2.10	0.000	0.000
CHIBA_RESPONSE_TO_TSA	38	2.10	0.000	0.000
DIRMEIER_LMP1_RESPONSE_EARLY	59	2.10	0.000	0.000
REACTOME_APC_C:CDH1_MEDIATED_DEGRADATION_OF_CDC20_AND_OTHER_APC_C:CDH1_TARGETED_PROTEINS_IN_LATE_MITOSIS_EARLY_G1	69	2.09	0.000	0.000
KEGG_RIBOSOME	82	2.08	0.000	0.000
REACTOME_CELLULAR_RESPONSES_TO_EXTERNAL_STIMULI	479	2.08	0.000	0.000
REACTOME_INTERLEUKIN_1_FAMILY_SIGNALING	121	2.08	0.000	0.000
REACTOME_CELLULAR_RESPONSES_TO_STRESS	396	2.08	0.000	0.000
HARRIS_HYPOXIA	67	2.07	0.000	0.000
REACTOME_ACTIVATION_OF_APC_C_AND_APC_C:CDC20_MEDIATED_DEGRADATION_OF_MITOTIC_PROTEINS	72	2.06	0.000	0.000
REACTOME_ANTIGEN_PROCESSING:_UBIQUITINATION_PROTEASOME_DEGRADATION	284	2.05	0.000	0.000
SMIRNOV_RESPONSE_TO_IR_2HR_UP	51	2.05	0.000	0.000
WONG_PROTEASOME_GENE_MODULE	50	2.03	0.000	0.001
REACTOME_CDK_MEDIATED_PHOSPHORYLATION_AND_REMOVAL_OF_CDC6	68	2.03	0.000	0.001
REACTOME_CLASS_I_MHC_MEDIATED_ANTIGEN_PROCESSING_PRESENTATION	342	2.03	0.000	0.001
REACTOME_CARBOXYTERMINAL_POST_TRANSLATIONAL_MODIFICATIONS_OF_TUBULIN	34	2.03	0.000	0.001
SESTO_RESPONSE_TO_UV_C0	99	2.03	0.000	0.001
REACTOME_FC_EPSILON_RECEPTOR_FCERI_SIGNALING	127	2.03	0.000	0.001
NAGASHIMA_EGF_SIGNALING_UP	51	2.02	0.000	0.001
AMIT_EGF_RESPONSE_40_HELA	38	2.02	0.000	0.001
REACTOME_DISORDERS_OF_TRANSMEMBRANE_TRANSPORTERS	152	2.01	0.000	0.001
REACTOME_ASSEMBLY_AND_CELL_SURFACE_PRESENTATION_OF_NMDA_RECEPTORS	39	2.01	0.000	0.001

KRIEG_HYPOXIA_VIA_KDM3A	46	2.01	0.000	0.001
UDAYAKUMAR_MED1_TARGETS_DN	224	2.01	0.000	0.001
SUH_COEXPRESSED_WITH_ID1_AND_ID2_UP	16	2.00	0.000	0.001
WINTER_HYPOXIA_METAGENE	208	2.00	0.000	0.001
REACTOME_UB_SPECIFIC_PROCESSING_PROTEASES	183	2.00	0.000	0.001
REACTOME_TCR_SIGNALING	107	2.00	0.000	0.001
GRAHAM_CML_QUIESCENT_VS_NORMAL_DIVIDING_UP	44	1.99	0.000	0.001
REACTOME_NONSENSE_MEDIATED_DECAY_NMD_INDEPENDENT_OF_THE_EXON_JUNCTION_COMPLEX_EJC	90	1.99	0.000	0.001
RASHI_RESPONSE_TO_IONIZING_RADIATION_1	36	1.98	0.000	0.001
REACTOME_GAP_JUNCTION_ASSEMBLY	26	1.98	0.000	0.001
REACTOME_AUTOPHAGY	104	1.98	0.000	0.001
REACTOME_REGULATION_OF_HSF1_MEDIATED_HEAT_SHOCK_RESPONSE	78	1.98	0.000	0.001
MARKEY_RB1_CHRONIC_LOF_DN	95	1.98	0.000	0.001
GARGALOVIC_RESPONSE_TO_OXIDIZED_PHOSPHOLIPIDS_YELLOW_UP	30	1.97	0.000	0.001
DAZARD_UV_RESPONSE_CLUSTER_G4	16	1.97	0.000	0.001
APPIERTO_RESPONSE_TO_FENRETINIDE_UP	31	1.97	0.000	0.001
KIM_WT1_TARGETS_8HR_UP	154	1.96	0.000	0.002
NAKAYAMA_FRA2_TARGETS	40	1.96	0.000	0.002
REACTOME_TRANSPORT_OF_CONNEXONS_TO_THE_PLASMA_MEMBRANE	19	1.96	0.000	0.002
PID_FRA_PATHWAY	31	1.96	0.000	0.002
RHEIN_ALL_GLUCOCORTICOID_THERAPY_UP	61	1.95	0.000	0.002
REACTOME_PCP_CE_PATHWAY	87	1.95	0.000	0.002
YAO_TEMPORAL_RESPONSE_TO_PROGESTERONE_CLUSTER_1	56	1.95	0.002	0.002
YAN_ESCAPE_FROM_ANOIKIS	20	1.95	0.000	0.002
REACTOME_PROGRAMMED_CELL_DEATH	166	1.95	0.000	0.002
REACTOME_REGULATED_NECROSIS	20	1.95	0.000	0.002
REACTOME_CYCLIN_A:CDK2_ASSOCIATED_EVENTS_AT_S_PHASE_ENTRY	83	1.95	0.000	0.002
DORSAM_HOXA9_TARGETS_DN	29	1.95	0.000	0.002
REACTOME_REGULATION_OF_MITOTIC_CELL_CYCLE	82	1.94	0.000	0.002
REACTOME_COPI_MEDIATED_ANTEROGRADE_TRANSPORT	97	1.94	0.000	0.002
REACTOME_MITOPHAGY	26	1.94	0.000	0.002
REACTOME_INTERLEUKIN_4_AND_INTERLEUKIN_13_SIGNALING	87	1.94	0.000	0.002
DAZARD_UV_RESPONSE_CLUSTER_G24	16	1.94	0.002	0.002
REACTOME_SIGNALING_BY_ROBO_RECEPTORS	202	1.93	0.000	0.002
DEN_INTERACT_WITH_LCA5	25	1.93	0.002	0.002
REACTOME_IRON_UPTAKE_AND_TRANSPORT	53	1.92	0.000	0.003
REACTOME_DEUBIQUITINATION	255	1.92	0.000	0.003
ZHANG_RESPONSE_TO_IKK_INHIBITOR_AND_TNF_UP	194	1.92	0.000	0.003
REACTOME_NONSENSE_MEDIATED_DECAY_NMD	110	1.92	0.000	0.003
REACTOME_TRANSCRIPTIONAL_REGULATION_BY_RUNX2	108	1.92	0.000	0.003
SARRIO_EPITHELIAL_MESENCHYMAL_TRANSITION_DN	129	1.92	0.000	0.003

REACTOME_SIGNALING_BY_THE_B_CELL_RECEPTOR_BCR	105	1.92	0.000	0.003
BILANGES_SERUM_AND_RAPAMYCIN_SENSITIVE_GENES	46	1.92	0.000	0.003
DAUER_STAT3_TARGETS_UP	39	1.92	0.002	0.003
AMIT_DELAYED_EARLY_GENES	18	1.92	0.000	0.003
DEBIASI_APOPTOSIS_BY_REOVIRUS_INFECTION_UP	275	1.91	0.000	0.003
REACTOME_RUNX1_REGULATES_TRANSCRIPTION_OF_GENES_INVOLVED_IN_DIFFEREN TIATION_OF_HSCS	108	1.91	0.000	0.003
AMIT_SERUM_RESPONSE_120_MCF10A	65	1.91	0.000	0.003
AMIT_SERUM_RESPONSE_40_MCF10A	29	1.90	0.000	0.003
REACTOME_PINK_PARKIN_MEDIATED_MITOPHAGY	19	1.90	0.000	0.003
AMIT_EGF_RESPONSE_60_HELA	41	1.90	0.000	0.003
WENG_POR_DOSAGE	15	1.90	0.000	0.003
REACTOME_ACTIVATION_OF_THE_MRNA_UPON_BINDING_OF_THE_CAP_BINDING_COMP LEX_AND_EIFS_AND_SUBSEQUENT_BINDING_TO_43S	57	1.90	0.000	0.003
FALVELLA_SMOKERS_WITH_LUNG_CANCER	70	1.90	0.000	0.003
REACTOME_PTEN_REGULATION	132	1.90	0.000	0.003
ABRAHAM_ALPC_VS_MULTIPLE_MYELOMA_UP	22	1.90	0.002	0.003
VARELA_ZMPSTE24_TARGETS_UP	40	1.90	0.000	0.003
REACTOME_ASSEMBLY_OF_THE_PRE_REPLICATIVE_COMPLEX	65	1.90	0.000	0.003
MCDOWELL_ACUTE_LUNG_INJURY_UP	37	1.89	0.000	0.004
REACTOME_MITOTIC_G2_G2_M_PHASES	193	1.89	0.000	0.004
REACTOME_ORC1_REMOVAL_FROM_CHROMATIN	67	1.89	0.000	0.004
GENTILE_UV_HIGH_DOSE_UP	18	1.89	0.000	0.004
REACTOME_HOST_INTERACTIONS_OF_HIV_FACTORS	121	1.88	0.000	0.004
REACTOME_MAP3K8_TPL2_DEPENDENT_MAPK1_3_ACTIVATION	16	1.88	0.000	0.004
SEMENZA_HIF1_TARGETS	33	1.88	0.000	0.004
BAKKER_FOXO3_TARGETS_UP	50	1.87	0.002	0.005
ZWANG_CLASS_2_TRANSIENTLY_INDUCED_BY_EGF	38	1.87	0.000	0.005
LIAN_LIPA_TARGETS_6M	51	1.87	0.000	0.005
ENK_UV_RESPONSE_KERATINOCYTE_UP	458	1.87	0.000	0.005
REACTOME_POST_CHAPERONIN_TUBULIN_FOLDING_PATHWAY	22	1.87	0.004	0.005
CAFFAREL_RESPONSE_TO_THC_UP	32	1.87	0.000	0.005
HAN_JNK_SINGALING_UP	29	1.86	0.000	0.005
REACTOME_INFECTIOUS_DISEASE	356	1.86	0.000	0.005
GRAHAM_NORMAL_QUIESCENT_VS_NORMAL_DIVIDING_UP	49	1.86	0.000	0.005
PRAMOONJAGO_SOX4_TARGETS_UP	49	1.86	0.000	0.006
LIAN_LIPA_TARGETS_3M	42	1.86	0.000	0.006
ODONNELL_TARGETS_OF_MYC_AND_TFRC_UP	71	1.86	0.000	0.006
REACTOME_GAP_JUNCTION_TRAFFICKING_AND_REGULATION	39	1.85	0.000	0.006
REACTOME_FORMATION_OF_TUBULIN_FOLDING_INTERMEDIATES_BY_CCT_TRIC	25	1.85	0.002	0.006
LAMB_CCND1_TARGETS	17	1.85	0.000	0.006
FERRARI_RESPONSE_TO_FENRETINIDE_UP	20	1.84	0.000	0.007

REACTOME_MACROAUTOPHAGY	86	1.84	0.000	0.007
REACTOME_NEDDYLATION	219	1.84	0.000	0.007
MISSIAGLIA_REGULATED_BY_METHYLATION_UP	108	1.83	0.000	0.007
GARGALOVIC_RESPONSE_TO_OXIDIZED_PHOSPHOLIPIDS_GREY_UP	17	1.83	0.006	0.008
PETROVA_ENDOTHELIUM_LYMPHATIC_VS_BLOOD_DN	138	1.83	0.000	0.008
RASHI_RESPONSE_TO_IONIZING_RADIATION_2	103	1.83	0.000	0.008
REACTOME_GOLGI_TO_ER_RETROGRADE_TRANSPORT	125	1.83	0.000	0.008
REACTOME_ACTIVATION_OF_AMPK_DOWNSTREAM_OF_NMDARS	26	1.83	0.000	0.008
TIAN_TNF_SIGNALING_NOT_VIA_NFKB	22	1.82	0.002	0.008
PHONG_TNF_TARGETS_UP	58	1.82	0.000	0.009
ZWANG_CLASS_1_TRANSIENTLY_INDUCED_BY_EGF	434	1.82	0.000	0.009
WIERENGA_STAT5A_TARGETS_GROUP2	45	1.82	0.000	0.009
KIM_WT1_TARGETS_12HR_UP	146	1.81	0.000	0.009
PELLICCIOTTA_HDAC_IN_ANTIGEN_PRESENTATION_UP	59	1.81	0.000	0.009
REACTOME_INFLUENZA_INFECTION	147	1.81	0.000	0.010
REACTOME_MITOTIC_METAPHASE_AND_ANAPHASE	191	1.81	0.000	0.010
GROSS_HYPOXIA_VIA_ELK3_ONLY_UP	25	1.81	0.002	0.010

Supplementary Table S6.

Significantly downregulated gene sets in MM.1S cells treated with combination treatment of PTC596 with bortezomib.

Gene set enrichment analysis using our RNA-seq data identified gene sets, which were significantly downregulated in MM.1S cells treated with the combination treatment versus control. (FDR w-value <0.01).

Hallmark

NAME	SIZE	NES	NOM p-val	FDR q-val
HALLMARK_E2F_TARGETS	196	-2.48	0.000	0.000
HALLMARK_G2M_CHECKPOINT	189	-1.88	0.000	0.001
HALLMARK_CHOLESTEROL_HOMEOSTASIS	72	-1.73	0.004	0.007

c2.all.v7.0

NAME	SIZE	NES	NOM p-val	FDR q-val
DUTERTRE_ESTRADIOL_RESPONSE_24HR_UP	303	-2.64	0.000	0.000
ROSTY_CERVICAL_CANCER_PROLIFERATION_CLUSTER	135	-2.55	0.000	0.000
VERNELL_RETINOBLASTOMA_PATHWAY_UP	69	-2.52	0.000	0.000
CONCANNON_APOPTOSIS_BY_EPOXOMICIN_DN	156	-2.49	0.000	0.000
ZHOU_CELL_CYCLE_GENES_IN_IR_RESPONSE_6HR	79	-2.49	0.000	0.000
MISSIAGLIA_REGULATED_BY_METHYLATION_DN	115	-2.42	0.000	0.000
GRAHAM_NORMAL_QUIESCENT_VS_NORMAL_DIVIDING_DN	85	-2.42	0.000	0.000
GRAHAM_CML_DIVIDING_VS_NORMAL_QUIESCENT_UP	169	-2.42	0.000	0.000
RUIZ_TNC_TARGETS_DN	140	-2.41	0.000	0.000
ZHOU_CELL_CYCLE_GENES_IN_IR_RESPONSE_24HR	116	-2.40	0.000	0.000
KOBAYASHI_EGFR_SIGNALING_24HR_DN	244	-2.37	0.000	0.000
ISHIDA_E2F_TARGETS	50	-2.36	0.000	0.000
SONG_TARGETS_OF_IE86_CMV_PROTEIN	54	-2.36	0.000	0.000
BURTON_ADIPOGENESIS_3	96	-2.34	0.000	0.000
FUJII_YBX1_TARGETS_DN	187	-2.33	0.000	0.000
REN_BOUND_BY_E2F	60	-2.32	0.000	0.000
ALCALAY_AML_BY_NPM1_LOCALIZATION_DN	169	-2.32	0.000	0.000
PUJANA_XPRSS_INT_NETWORK	161	-2.30	0.000	0.000
PUJANA_BRCA2_PCC_NETWORK	403	-2.30	0.000	0.000
PUJANA_BRCA_CENTERED_NETWORK	116	-2.26	0.000	0.000
WHITEFORD_PEDIATRIC_CANCER_MARKERS	111	-2.25	0.000	0.000
FISCHER_G1_S_CELL_CYCLE	188	-2.25	0.000	0.000
CROONQUIST_IL6_DEPRIVATION_DN	93	-2.23	0.000	0.000
REACTOME_DNA_STRAND_ELONGATION	32	-2.23	0.000	0.000
KAUFFMANN_MELANOMA_RELAPSE_UP	61	-2.22	0.000	0.000
BLUM_RESPONSE_TO_SALIRASIB_DN	320	-2.22	0.000	0.000
KEGG_DNA_REPLICATION	36	-2.20	0.000	0.000

AFFAR_YY1_TARGETS_DN	197	-2.20	0.000	0.000
SCHMIDT_POR_TARGETS_IN_LIMB_BUD_UP	24	-2.20	0.000	0.000
PYEON_HPV_POSITIVE_TUMORS_UP	86	-2.19	0.000	0.000
FOURNIER_ACINAR_DEVELOPMENT_LATE_2	273	-2.18	0.000	0.000
STEIN_ESRRA_TARGETS_RESPONSIVE_TO_ESTROGEN_DN	39	-2.18	0.000	0.000
SARRIO_EPITHELIAL_MESENCHYMAL_TRANSITION_UP	175	-2.17	0.000	0.000
CHICAS_RB1_TARGETS_GROWING	214	-2.16	0.000	0.000
KONG_E2F3_TARGETS	91	-2.15	0.000	0.000
REACTOME_CHOLESTEROL_BIOSYNTHESIS	24	-2.15	0.000	0.000
WENG_POR_TARGETS_GLOBAL_UP	16	-2.14	0.000	0.000
LEE_EARLY_T_LYMPHOCYTE_UP	99	-2.14	0.000	0.000
PUJANA_BREAST_CANCER_WITH_BRCA1_MUTATED_UP	54	-2.14	0.000	0.000
MARKEY_RB1_ACUTE_LOF_UP	214	-2.13	0.000	0.000
REACTOME_EXTENSION_OF_TELOMERES	30	-2.13	0.000	0.000
MITSIADES_RESPONSE_TO_APLIDIN_DN	241	-2.12	0.000	0.000
MARKEY_RB1_CHRONIC_LOF_UP	98	-2.11	0.000	0.000
CHICAS_RB1_TARGETS_SENESCENT	488	-2.10	0.000	0.000
BILD_E2F3_ONCOGENIC_SIGNATURE	223	-2.10	0.000	0.000
REACTOME_ACTIVATION_OF_THE_PRE_REPLICATIVE_COMPLEX	33	-2.09	0.000	0.000
REACTOME_ACTIVATION_OF_ATR_IN_RESPONSE_TO_REPLICATION_STRESS	37	-2.09	0.000	0.000
MANALO_HYPOXIA_DN	283	-2.09	0.000	0.000
FRASOR_RESPONSE_TO_SERM_OR_FULVESTRANT_DN	45	-2.09	0.000	0.000
MORI_IMMATURE_B_LYMPHOCYTE_DN	86	-2.09	0.000	0.000
SCHUHMACHER_MYC_TARGETS_UP	78	-2.07	0.000	0.000
BENPORATH_ES_2	35	-2.07	0.000	0.000
MOLENAAR_TARGETS_OF_CCND1_AND_CDK4_DN	52	-2.07	0.000	0.000
CHIANG_LIVER_CANCER_SUBCLASS_PROLIFERATION_UP	163	-2.07	0.000	0.000
CUI_TCF21_TARGETS_2_UP	352	-2.07	0.000	0.000
KANG_DOXORUBICIN_RESISTANCE_UP	52	-2.06	0.000	0.000
CROONQUIST_NRAS_SIGNALING_DN	69	-2.06	0.000	0.000
REACTOME_HDR_THROUGH_SINGLE_STRAND_ANNEALING_SSA	36	-2.05	0.000	0.000
FERRANDO_HOX11_NEIGHBORS	17	-2.05	0.000	0.000
FLORIO_NEOCORTEX_BASAL_RADIAL_GLIA_DN	166	-2.05	0.000	0.000
WHITFIELD_CELL_CYCLE_LITERATURE	41	-2.05	0.000	0.000
MORI_EMU_MYC_LYMPHOMA_BY_ONSET_TIME_UP	102	-2.05	0.000	0.000
GAL_LEUKEMIC_STEM_CELL_DN	173	-2.05	0.000	0.000
LE_EGR2_TARGETS_UP	101	-2.05	0.000	0.000
SOTIRIOU_BREAST_CANCER_GRADE_1_VS_3_UP	147	-2.04	0.000	0.000
BURTON_ADIPOGENESIS_PEAK_AT_16HR	38	-2.04	0.000	0.000
KAMMINGA_EZH2_TARGETS	41	-2.04	0.000	0.000
EGUCHI_CELL_CYCLE_RB1_TARGETS	23	-2.04	0.000	0.000

SMID_BREAST_CANCER_RELAPSE_IN_BRAIN_UP	27	-2.03	0.000	0.000
SENGUPTA_NASOPHARYNGEAL_CARCINOMA_UP	276	-2.03	0.000	0.000
REACTOME_HOMOLOGOUS_DNA_PAIRING_AND_STRAND_EXCHANGE	41	-2.03	0.000	0.000
REACTOME_TELOMERE_C_STRAND_LAGGING_STRAND_SYNTHESIS	24	-2.03	0.000	0.000
SUNG_METASTASIS_STROMA_DN	47	-2.03	0.000	0.000
WHITFIELD_CELL_CYCLE_G1_S	125	-2.01	0.000	0.000
TAKEDA_TARGETS_OF_NUP98_HOXA9_FUSION_16D_UP	138	-2.01	0.000	0.000
GARGALOVIC_RESPONSE_TO_OXIDIZED_PHOSPHOLIPIDS_TURQUOISE_DN	53	-2.00	0.000	0.001
ZHAN_MULTIPLE_MYELOMA_PR_UP	43	-2.00	0.000	0.001
TORCHIA_TARGETS_OF_EWSR1_FLI1_FUSION_DN	271	-2.00	0.000	0.001
REACTOME_FATTY_ACYL_COA_BIOSYNTHESIS	34	-1.98	0.000	0.001
REACTOME_REGULATION_OF_CHOLESTEROL_BIOSYNTHESIS_BY_SREBP_SREBF	55	-1.98	0.000	0.001
REACTOME_ACTIVATION_OF_GENE_EXPRESSION_BY_SREBF_SREBP	42	-1.98	0.000	0.001
BOYAULT_LIVER_CANCER_SUBCLASS_G23_UP	52	-1.98	0.000	0.001
DUTERTRE_ESTRADIOL_RESPONSE_6HR_UP	214	-1.98	0.000	0.001
KEGG_BUTANOATE_METABOLISM	29	-1.98	0.000	0.001
ODONNELL_TFRC_TARGETS_DN	120	-1.97	0.000	0.001
STEIN_ESR1_TARGETS	73	-1.97	0.000	0.001
HORTON_SREBF_TARGETS	23	-1.97	0.000	0.001
YU_MYC_TARGETS_UP	40	-1.97	0.000	0.001
BENPORATH_ES_1	341	-1.97	0.000	0.001
HOFFMANN_LARGE_TO_SMALL_PRE_BII_LYMPHOCYTE_UP	142	-1.97	0.000	0.001
REACTOME_PCNA_DEPENDENT_LONG_PATCH_BASE_EXCISION_REPAIR	21	-1.95	0.002	0.001
VALK_AML_CLUSTER_10	24	-1.95	0.000	0.001
SHEDDEN_LUNG_CANCER_POOR_SURVIVAL_A6	428	-1.94	0.000	0.001
KEGG_BIOSYNTHESIS_OF_UNSATURATED_FATTY_ACIDS	18	-1.94	0.000	0.001
BIOCARTA_MCM_PATHWAY	18	-1.93	0.000	0.002
JAATINEN_HEMATOPOIETIC_STEM_CELL_UP	272	-1.93	0.000	0.002
BURTON_ADIPOGENESIS_PEAK_AT_24HR	38	-1.93	0.000	0.002
RHEIN_ALL_GLUCOCORTICOID_THERAPY_DN	350	-1.93	0.000	0.002
KYNG_WERNER_SYNDROM_DN	17	-1.93	0.000	0.002
MORI_LARGE_PRE_BII_LYMPHOCYTE_UP	81	-1.93	0.000	0.002
HORIUCHI_WTAP_TARGETS_DN	294	-1.92	0.000	0.002
PAL_PRMT5_TARGETS_UP	179	-1.92	0.000	0.002
VILLANUEVA_LIVER_CANCER_KRT19_UP	164	-1.92	0.000	0.002
KEGG_STEROID_BIOSYNTHESIS	17	-1.92	0.000	0.002
REACTOME_LAGGING_STRAND_SYNTHESIS	20	-1.92	0.000	0.002
REACTOME_RESOLUTION_OF_D_LOOP_STRUCTURES	31	-1.92	0.000	0.002
LI_WILMS_TUMOR_VS_FETAL_KIDNEY_1_DN	155	-1.91	0.000	0.002
REACTOME_RESOLUTION_OF_AP_SITES_VIA_THE_MULTIPLE_NUCLEOTI	25	-1.91	0.004	0.002

DE_PATCH_REPLACEMENT_PATHWAY				
WANG_RESPONSE_TO_GSK3_INHIBITOR_SB216763_DN	336	-1.90	0.000	0.002
MEISSNER_BRAIN_HCP_WITH_H3_UNMETHYLATED	25	-1.90	0.000	0.002
ZHAN_MULTIPLE_MYELOMA_CD1_VS_CD2_DN	47	-1.90	0.000	0.002
KEGG_MISMATCH_REPAIR	23	-1.90	0.000	0.002
PID_BARD1_PATHWAY	28	-1.90	0.000	0.002
TANG_SENESCENCE_TP53_TARGETS_DN	51	-1.90	0.000	0.002
REACTOME_METABOLISM_OF_STEROIDS	123	-1.90	0.000	0.002
SERVITJA_LIVER_HNF1A_TARGETS_DN	114	-1.90	0.000	0.002
VANHARANTA_UTERINE_FIBROID_UP	37	-1.90	0.000	0.002
MORI_MATURE_B_LYMPHOCYTE_DN	67	-1.89	0.000	0.002
LY_AGING_PREMATURE_DN	27	-1.89	0.002	0.002
REACTOME_RESOLUTION_OF_ABASIC_SITES_AP_SITES	38	-1.89	0.000	0.002
KRASNOSELSKAYA_ILF3_TARGETS_DN	38	-1.89	0.000	0.002
JACKSON_DNMT1_TARGETS_DN	15	-1.89	0.002	0.002
BENPORATH_PROLIFERATION	140	-1.89	0.000	0.003
REACTOME_CYTOSOLIC_SULFONATION_OF_SMALL_MOLECULES	17	-1.88	0.000	0.003
WINNEPENNINCKX_MELANOMA_METASTASIS_UP	156	-1.88	0.000	0.003
CHEN_ETV5_TARGETS_TESTIS	19	-1.88	0.000	0.003
SHAFFER_IRF4_TARGETS_IN_ACTIVATED_DENDRITIC_CELL	62	-1.88	0.000	0.003
KEGG_HEMATOPOIETIC_CELL_LINEAGE	54	-1.88	0.000	0.003
REACTOME_TELOMERE_MAINTENANCE	68	-1.88	0.000	0.003
REACTOME_CHROMOSOME_MAINTENANCE	92	-1.87	0.000	0.003
LI_INDUCED_T_TO_NATURAL_KILLER_DN	121	-1.87	0.000	0.003
REACTOME_RESOLUTION_OF_D_LOOP_STRUCTURES_THROUGH_SYNTHESIS_DEPENDENT_STRAND_ANNEALING_SDSA	25	-1.86	0.002	0.004
MORI_PRE_BI_LYMPHOCYTE_UP	74	-1.86	0.000	0.004
ZHANG_BREAST_CANCER_PROGENITORS_UP	396	-1.85	0.000	0.004
ODONNELL_TARGETS_OF_MYC_AND_TFRC_DN	43	-1.85	0.000	0.004
KIM_MYCN_AMPLIFICATION_TARGETS_UP	82	-1.84	0.000	0.005
LINDGREN_BLADDER_CANCER_CLUSTER_3_UP	306	-1.84	0.000	0.005
PETROVA_ENDOTHELIUM_LYMPHATIC_VS_BLOOD_UP	112	-1.83	0.000	0.006
REACTOME_MISMATCH_REPAIR	15	-1.83	0.000	0.006
KEGG_BASE_EXCISION_REPAIR	33	-1.83	0.000	0.006
PODAR_RESPONSE_TO_ADAPHOSTIN_DN	16	-1.83	0.002	0.006
WANG_CISPLATIN_RESPONSE_AND_XPC_UP	154	-1.83	0.000	0.006
KAUFFMANN_DNA_REPAIR_GENES	225	-1.82	0.000	0.007
VERHAAK_AML_WITH_NPM1_MUTATED_DN	200	-1.82	0.000	0.007
WONG_EMBRYONIC_STEM_CELL_CORE	325	-1.81	0.000	0.008
BOYLAN_MULTIPLE_MYELOMA_C_UP	43	-1.80	0.000	0.008
TOYOTA_TARGETS_OF_MIR34B_AND_MIR34C	417	-1.80	0.000	0.009
ZHENG_GLIOBLASTOMA_PLASTICITY_UP	209	-1.80	0.000	0.009

SCHAEFFER_SOX9_TARGETS_IN_PROSTATE_DEVELOPMENT_DN	40	-1.80	0.000	0.009
SLEBOS_HEAD_AND_NECK_CANCER_WITH_HPV_UP	72	-1.79	0.000	0.009
VECCHI_GASTRIC_CANCER_EARLY_UP	388	-1.79	0.000	0.010

Supplementary Table S7.

Enriched pathways and processes characteristic of upregulated genes in both PTC596 single and bortezomib single in our RNA-seq (FDR p <0.05, top 50).

term_id	term_name	p_value	negative_log10_ of _p_value
GO:0003735	structural constituent of ribosome	0.000	280.9
GO:0044391	ribosomal subunit	0.000	276.0
KEGG:03010	Ribosome	0.000	269.5
GO:0005840	ribosome	0.000	256.2
REAC:R-HSA-72766	Translation	0.000	203.7
GO:0005198	structural molecule activity	0.000	177.3
GO:0006412	translation	0.000	176.0
GO:0043043	peptide biosynthetic process	0.000	174.1
GO:0022626	cytosolic ribosome	0.000	174.0
GO:1990904	ribonucleoprotein complex	0.000	172.5
WP:WP477	Cytoplasmic Ribosomal Proteins	0.000	167.0
GO:0006518	peptide metabolic process	0.000	163.9
GO:0043604	amide biosynthetic process	0.000	163.9
REAC:R-HSA-156902	Peptide chain elongation	0.000	157.0
REAC:R-HSA-192823	Viral mRNA Translation	0.000	157.0
GO:0006614	SRP-dependent cotranslational protein targeting to membrane	0.000	155.0
GO:0015934	large ribosomal subunit	0.000	154.3
REAC:R-HSA-2408557	Selenocysteine synthesis	0.000	153.4
REAC:R-HSA-156842	Eukaryotic Translation Elongation	0.000	153.4
REAC:R-HSA-72764	Eukaryotic Translation Termination	0.000	153.4
REAC:R-HSA-975956	Nonsense Mediated Decay (NMD) independent of the Exon Junction Complex (EJC)	0.000	151.7
GO:0006613	cotranslational protein targeting to membrane	0.000	151.7
GO:0043603	cellular amide metabolic process	0.000	148.5
REAC:R-HSA-72689	Formation of a pool of free 40S subunits	0.000	147.3
**GO:0045047**	**protein targeting to ER**	**0.000**	**147.1**
**GO:0072599**	**establishment of protein localization to endoplasmic reticulum**	**0.000**	**145.1**
REAC:R-HSA-156827	L13a-mediated translational silencing of Ceruloplasmin expression	0.000	141.3
GO:0000184	nuclear-transcribed mRNA catabolic process, nonsense-mediated decay	0.000	141.0
REAC:R-HSA-72706	GTP hydrolysis and joining of the 60S ribosomal subunit	0.000	140.7
REAC:R-HSA-1799339	SRP-dependent cotranslational protein targeting to membrane	0.000	140.7
REAC:R-HSA-975957	Nonsense Mediated Decay (NMD) enhanced by the Exon Junction Complex (EJC)	0.000	139.1
REAC:R-HSA-927802	Nonsense-Mediated Decay (NMD)	0.000	139.1

REAC:R-HSA-2408522	Selenoamino acid metabolism	0.000	138.6
REAC:R-HSA-72737	Cap-dependent Translation Initiation	0.000	137.1
REAC:R-HSA-72613	Eukaryotic Translation Initiation	0.000	137.1
GO:0070972	protein localization to endoplasmic reticulum	0.000	135.1
GO:0044445	cytosolic part	0.000	131.9
REAC:R-HSA-168273	Influenza Viral RNA Transcription and Replication	0.000	131.4
REAC:R-HSA-168255	Influenza Life Cycle	0.000	128.0
REAC:R-HSA-168254	Influenza Infection	0.000	124.2
GO:0019083	viral transcription	0.000	123.4
GO:1901566	organonitrogen compound biosynthetic process	0.000	120.5
GO:0019080	viral gene expression	0.000	120.1
CORUM:306	Ribosome, cytoplasmic	0.000	119.9
GO:0006612	protein targeting to membrane	0.000	119.5
GO:0006413	translational initiation	0.000	118.8
REAC:R-HSA-9010553	Regulation of expression of SLITs and ROBOs	0.000	118.8
GO:0000956	nuclear-transcribed mRNA catabolic process	0.000	116.2
REAC:R-HSA-6791226	Major pathway of rRNA processing in the nucleolus and cytosol	0.000	115.7

Supplementary Table S8.

List of primers used for Quantitative RT-PCR.

Sequences of primers used for quantitative real time PCR.

Target Gene	Primers	Sequences (5' to 3')
BMI1	Forward	CGTGTATTGTTCGTTACCTGGA
	Reverse	TTCAGTAGTGGTCTGGTCTTGT
mBmi1	Forward	AAACCAGACCACTCCTGAACA
	Reverse	TCTTCTTCTCTTCATCTCATTTTTGA
DDIT3	Forward	AGAACCAGGAAACGGAAACAGA
	Reverse	TCTCCTTCATGCGCTGCTTT
HSPA5	Forward	CAATCAAGGTCTATGAAGGTGAAAGA
	Reverse	CACATCTATCTCAAAGGTGACTTCAATC
ATF4	Forward	CCCTTCACCTTCTTACAACCTC
	Reverse	TGCCCAGCTCTAAACTAAAGGA
GAPDH	Forward	CTGACTTCAACAGCGACACC
	Reverse	TAGCCAAATTCGTTGTCATACC
ACTB	Forward	GGATGCAGAAGGAGATCACTG
	Reverse	CGATCCACACGGAGTACTTG

Supplementary Figure S1.

A

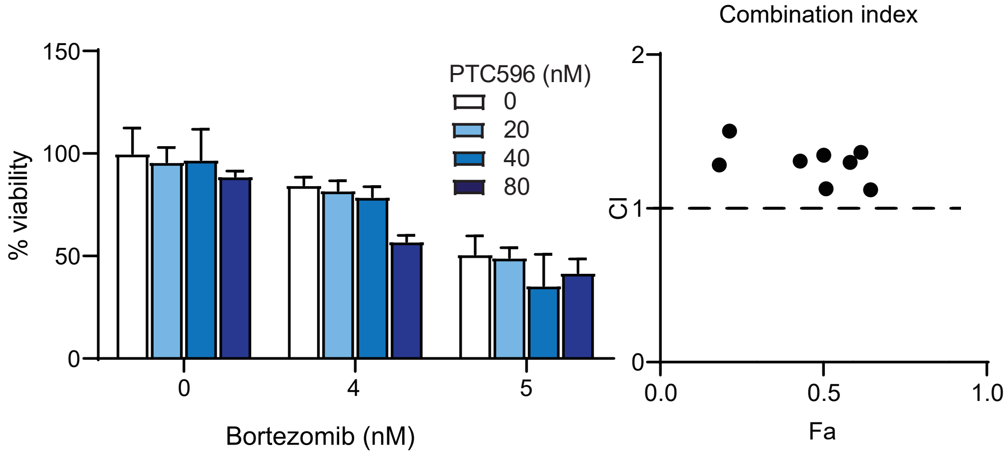

Supplementary Figure S1. PTC596 and bortezomib do not exert synergistic anti-MM activity without BMSCs. (A) MTS assay of MM.1S cells treated with the indicated doses of PTC596 for 48 hours and bortezomib for the last 24 hours. The y-axis presents percent viability relative to the untreated control. Data are shown as means ± SD of triplicate samples. Combination index values are shown in the right graph.

Supplementary Figure S2.

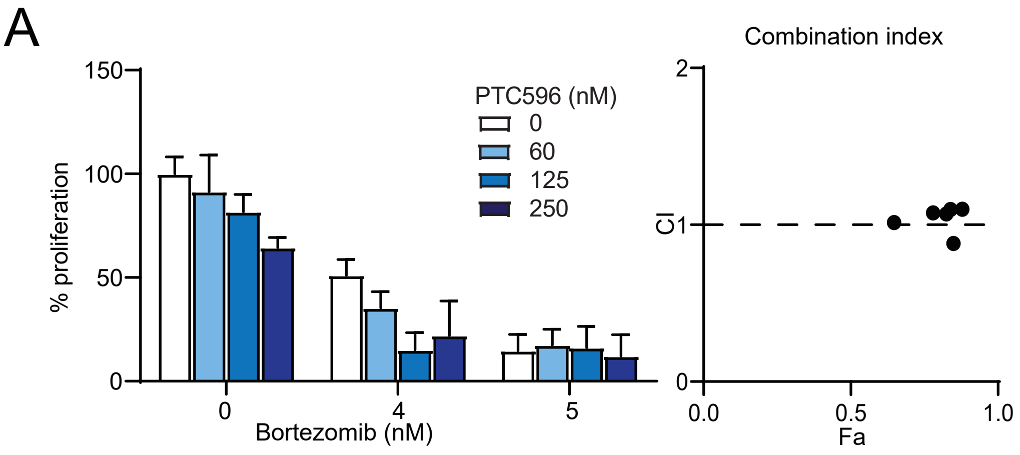

Supplementary Figure S2. PTC596 and bortezomib exert additive anti-MM activity when the cells are co-cultured with BMSCs

(A) BrdU proliferation assay of OPM-2 cells co-cultured with BMSCs derived from MM patients upon treatment with the indicated doses of PTC596 and bortezomib for 48 hours. Results of triplicate experiments and combination index values are shown in the left and right graphs, respectively.

Supplementary Figure S3.

A

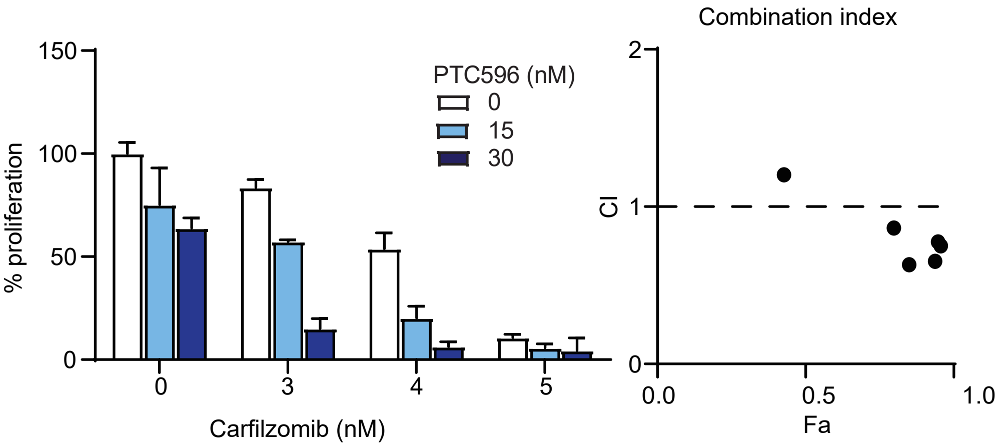

Supplementary Figure S3. PTC596 and carfilzomib exert synergistic anti-MM activity when the cells are co-cultured with BMSCs

(A) BrdU proliferation assay of MM.1S cells co-cultured with BMSCs derived from MM patients upon treatment with the indicated doses of PTC596 and carfilzomib for 48 hours. Results of triplicate experiments and combination index values are shown in the left and right graphs, respectively.

Supplementary Figure S4.

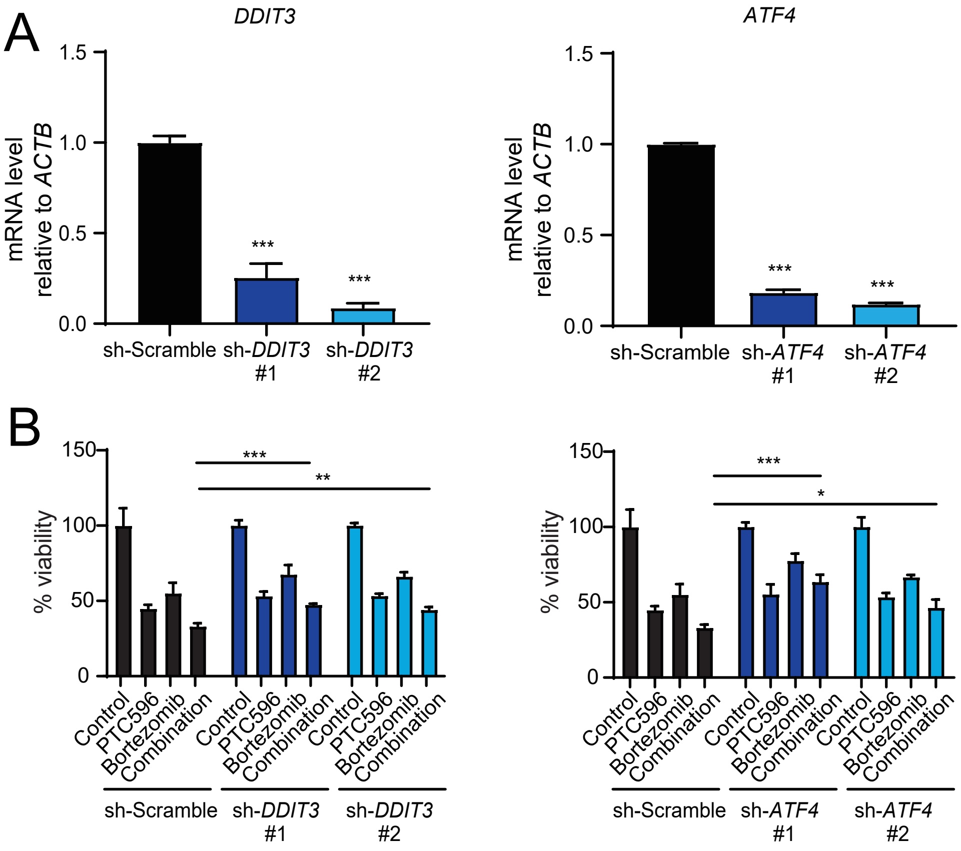

Supplementary Figure S4. ER stress pathway contributes to the cytotoxicity of the combination treatment. (A, B) RPMI8226 cells transduced with the indicated lentiviruses were selected by puromycin. Those were subjected to quantitative RT-PCR (A) and MTS assays (B). (A) Quantitative mRNA expression of *DDIT3* and *ATF4*. Y-axis represents fold change after normalization to *ACTB* and error bars represent SD of triplicates. ***P < 0.001 by one-way ANOVA. (B) MTS assays of RPMI8226 cells transduced with sh-*DDIT3* and sh-Scramble lentiviruses (left graphs), and RPMI8226 cells transduced with sh-*ATF4* and sh-Scramble lentiviruses (right graphs), treated with or without PTC596 (15 nM) in the presence or absence of bortezomib (6 nM) for 48 hours. The y-axis presents percent viability relative to the untreated control. Data are shown as means ± SD of triplicate samples. *P < 0.05; **P < 0.01; *** P < 0.001 using Student' s *t*-test.

Supplementary Figure S5.

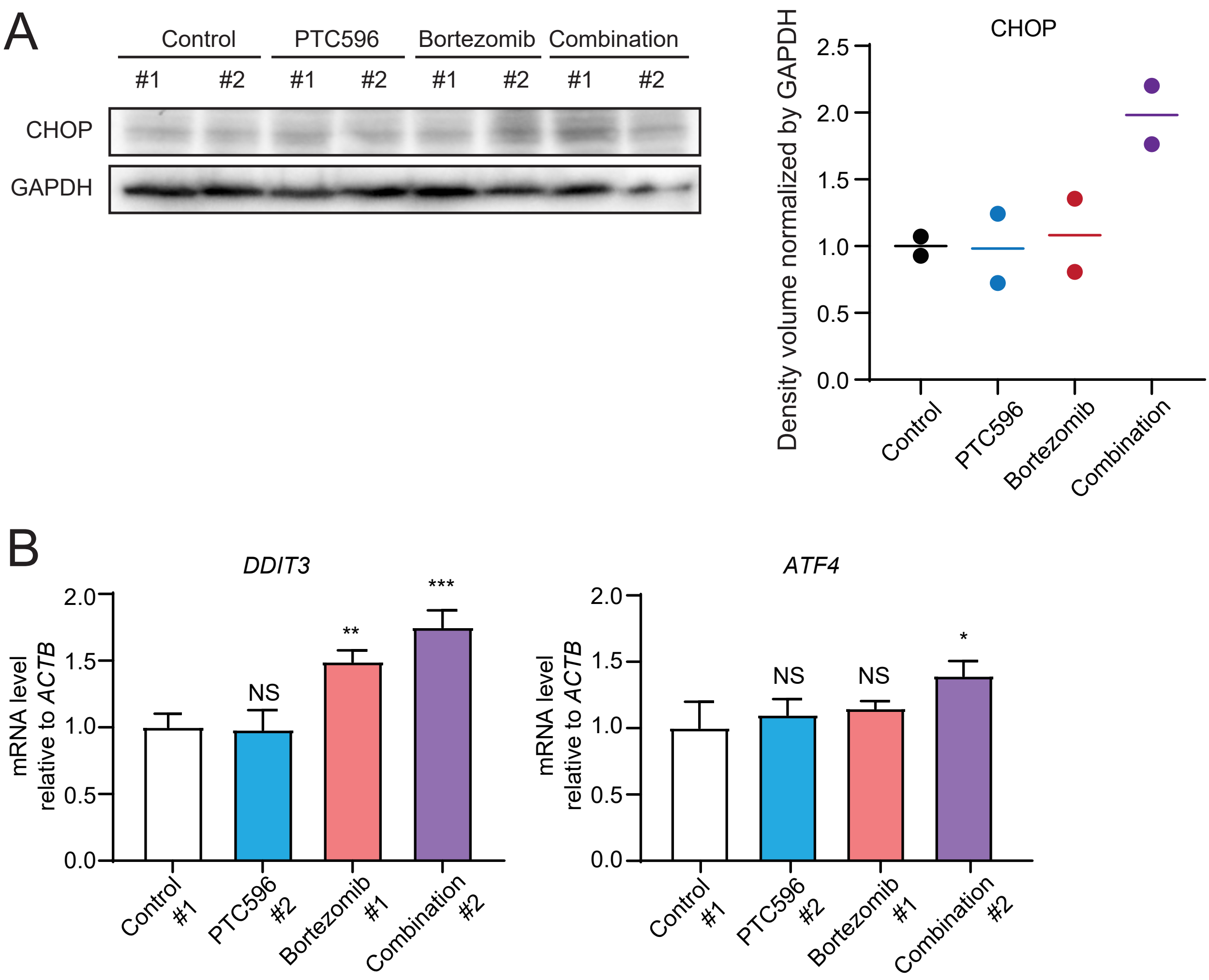

Supplementary Figure S5. Endoplasmic reticulum stress is augmented by the combination of PTC596 and bortezomib *in vivo*.

(A, B) Tumors harvested from mice treated for 2 weeks with oral PTC596 (6.25 mg/kg) twice a week, subcutaneous bortezomib (0.5 mg/kg) twice a week, or the combination were subjected to western blotting (A) and RT-PCR (B). (A) Western blotting analysis of the indicated proteins in MM tumor cells. GAPDH served as a loading control. The right graphs show the density volume normalized by GAPDH. (B) Quantitative RT-PCR of mRNA expression of *DDIT3* and *ATF4*. *ACTB* was used to normalize the amount of input RNA. Data are shown as mean ± SD (n=3). *P < 0.05; **P < 0.01; *** P < 0.001; ns, not significant using one-way ANOVA.Y-axis represents fold change after normalization to *ACTB*, and error bars represent SD of triplicates.

Supplementary Figure S6.

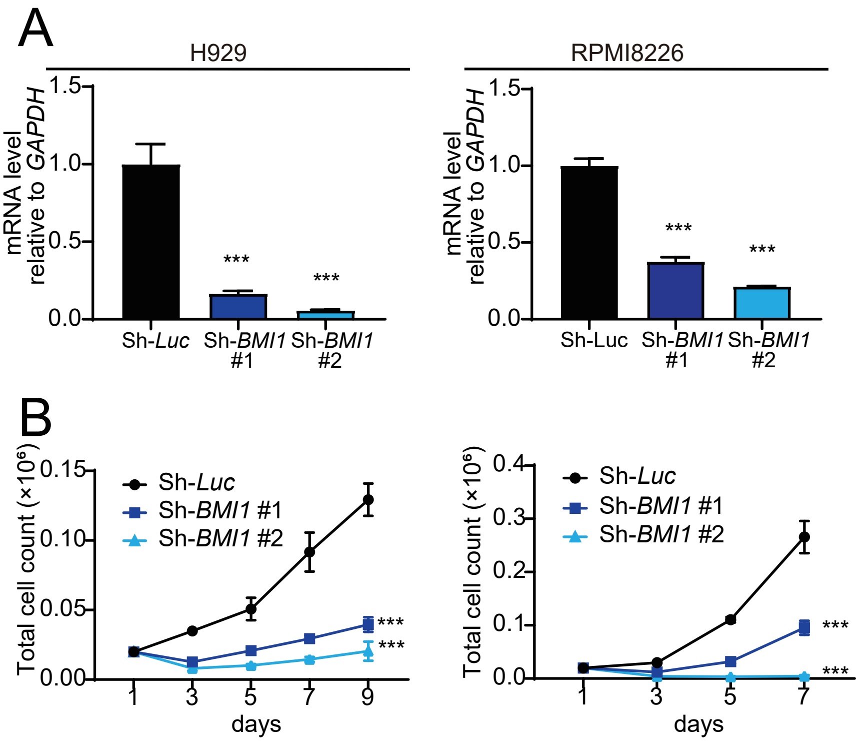

Supplementary Figure S6. MM cells depend on BMI1 for their growth.

(A, B) Human MM cell lines H929 and RPMI8226 cells transduced with the indicated lentiviruses were selected by cell sorting for GFP expression. Those were subjected to quantitative RT-PCR (A) and cell counting assay (B). (A) Quantitative RT-PCR of mRNA expression of *BMI1*. Y-axis represents fold change after normalization to *GAPDH*, and error bars represent SD of triplicates. ***P < 0.001 by one-way ANOVA. (B) Cell counting assay using trypan blue on the indicated days of cultures. Data represent mean ± SD of triplicate cultures. ***P < 0.001 by one-way ANOVA.

Whole blots for cropped images

Whole blot for cropped image for figure 2A

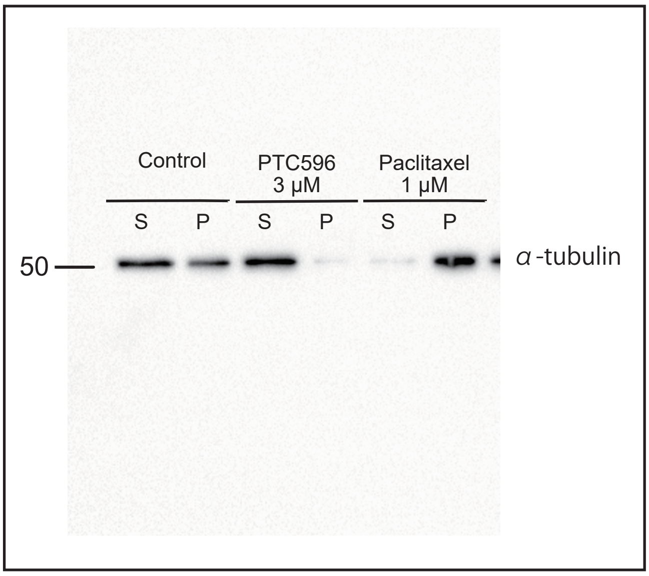

Whole blots for cropped images for figure 3C

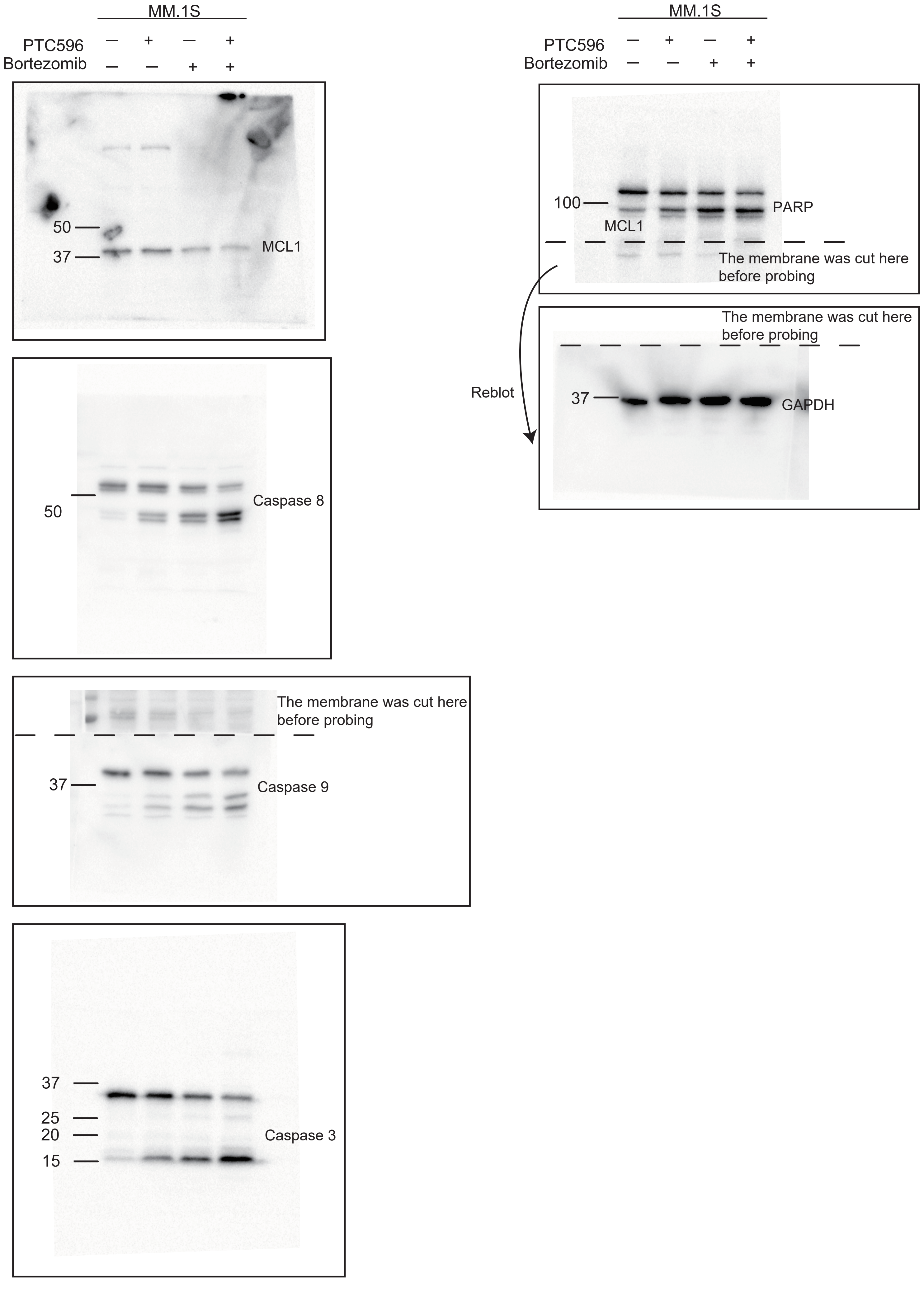

Whole blots for cropped images for figure 3C

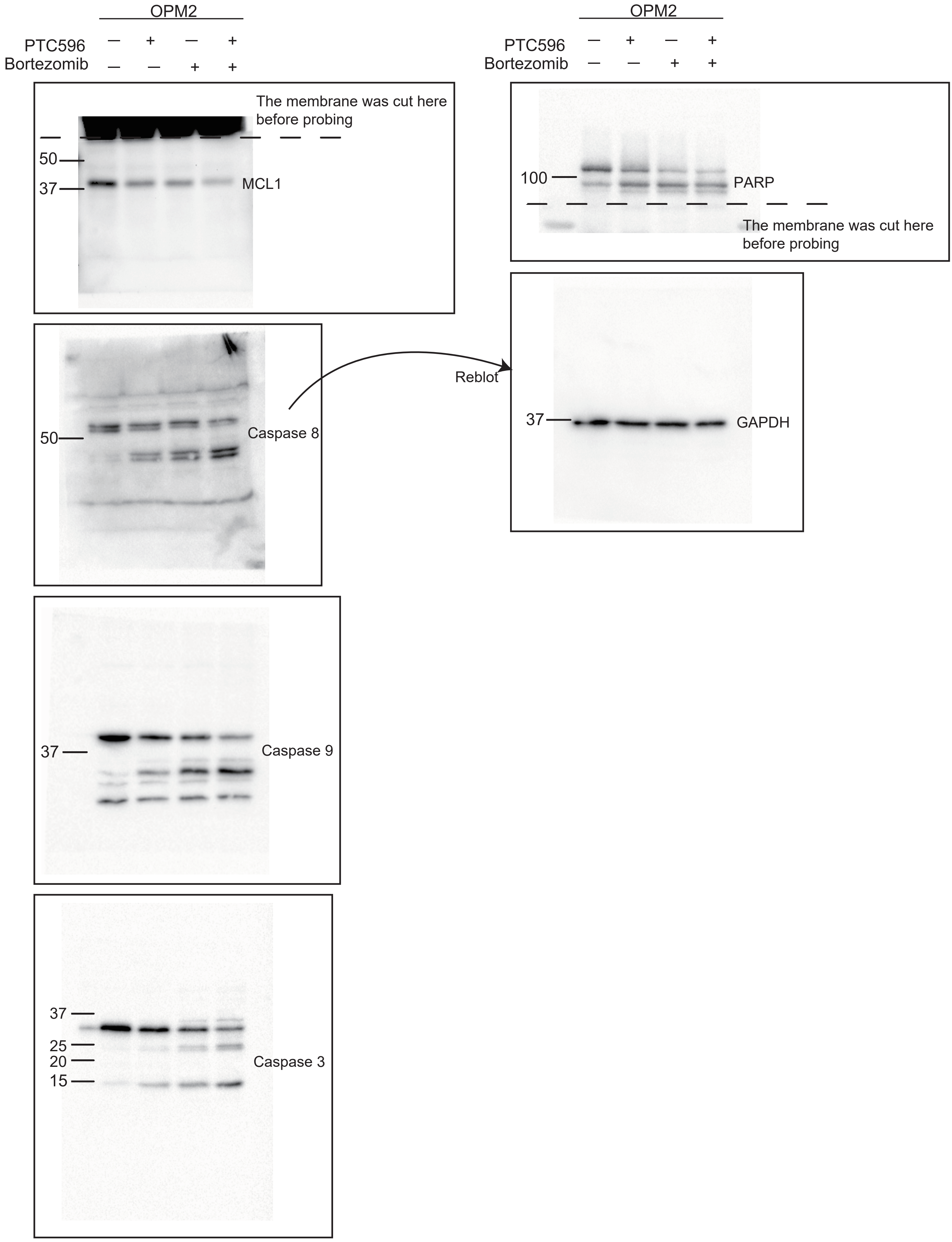

Whole blots for cropped images for figure 5B

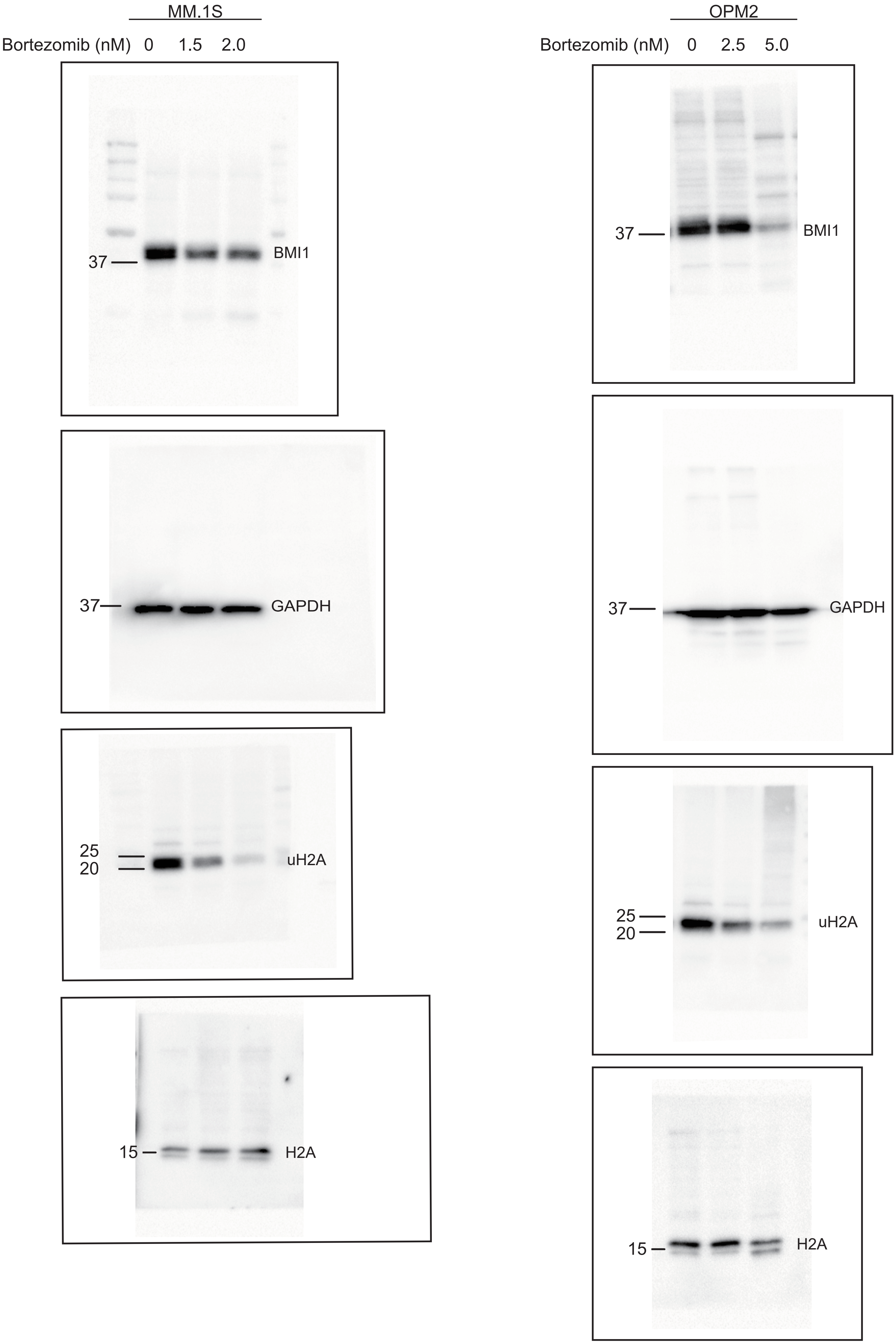

Whole blots for cropped images for figure 5C

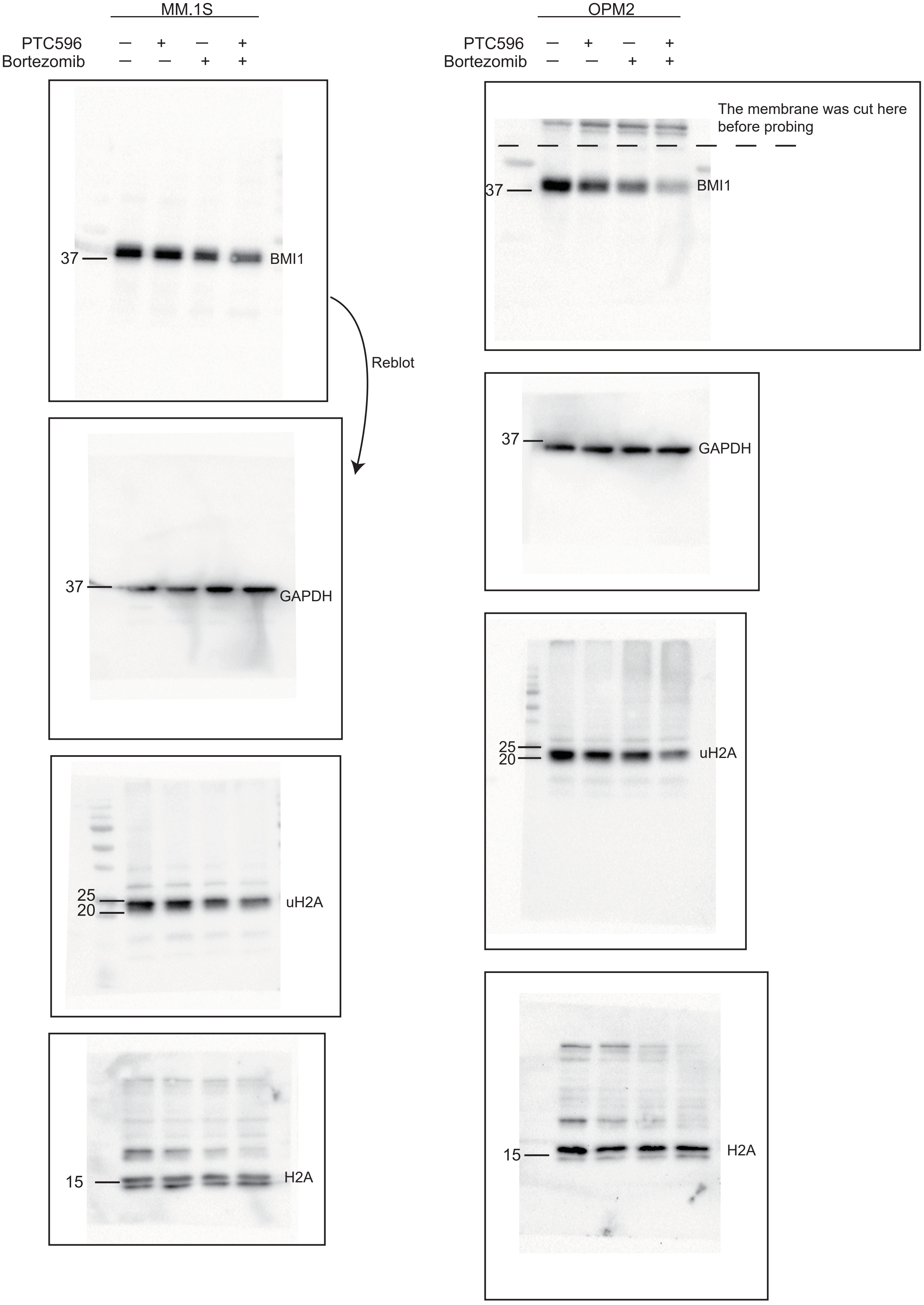

Whole blots for cropped images for figure 6C

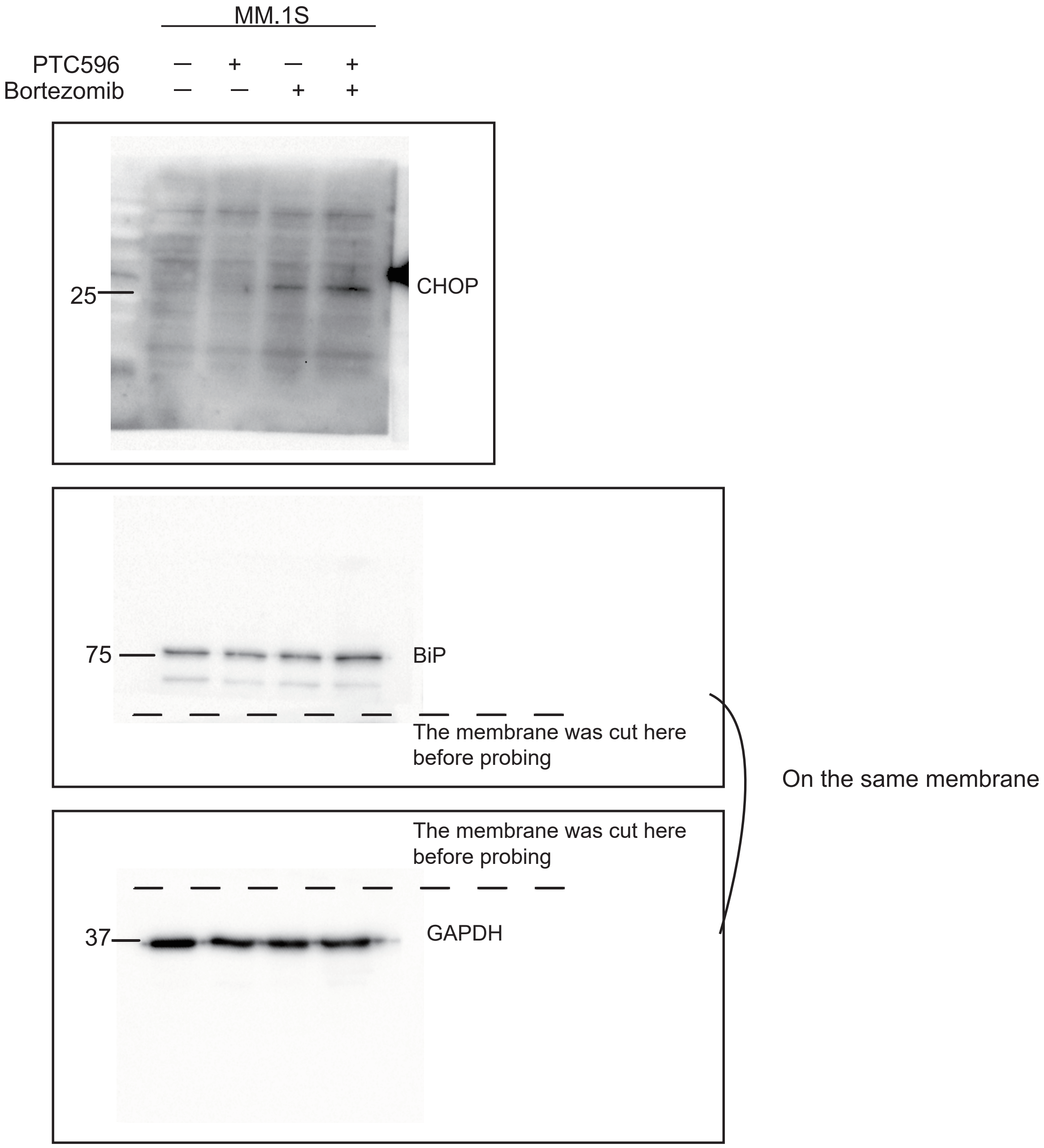

Whole blots for cropped images for supplementaly figure 5A

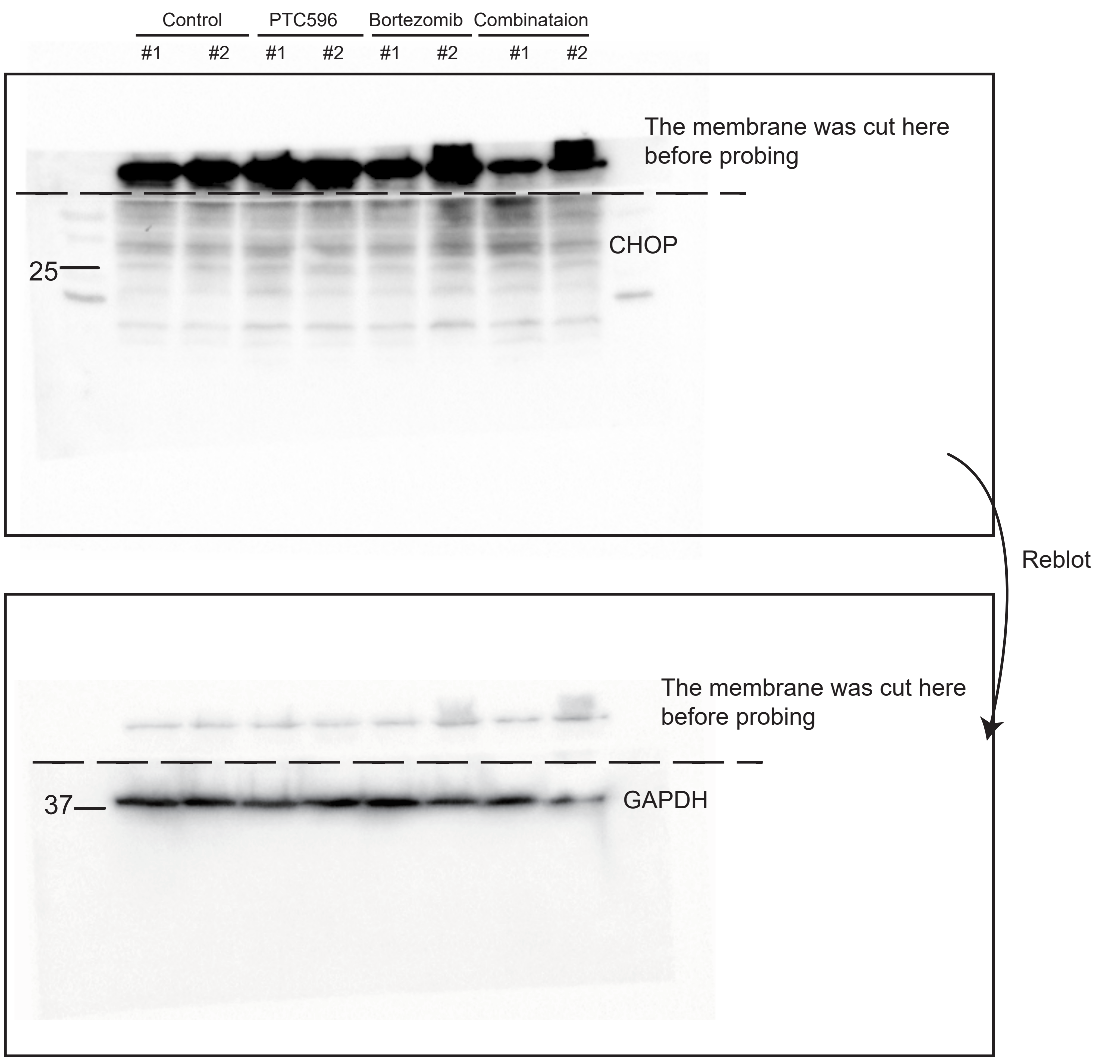

Supplement: Supplementary file 1 — Supplementary Information. [file 41598_2021_81577_MOESM1_ESM.pdf]
